# Insights from the co-creation process in communities of practice for urban water management

**DOI:** 10.12688/openreseurope.16604.1

**Published:** 2024-04-24

**Authors:** Marianne Wehbe, Manuel Bea, Elena Goicolea Güemez, Ulf Stein, Nicolas Caradot, Juan Goicolea, Elena Lopez Gunn, Gerardo Anzaldua

**Affiliations:** 1 Icatalist, Madrid, Spain; 2 Ecologic Institut gemeinnützige GmbH, Berlin, Germany; 3 Kompetenzzentrum Wasser Berlin gGmbH, Berlin, Berlin, Germany

**Keywords:** water sector; communities of practice; co-creation process; digital solutions

## Abstract

This article presents a practical case of a co-creation process within the framework of the European project Digital Water City (DWC). DWC sought to address the context and challenges of water in Europe with a participatory approach of co-creation as an integral part of Communities of Practice. To situate the concepts within their theoretical framework, the different approximations of co-creation and Communities of Practice are examined. The article analyzes the ways in which they have been used and applied in DWC as well as the ways in which the project has managed to articulate them at different levels. It discusses what has been done so far, whether the objectives related to co-creation have been achieved, what its added value to the project was, and the possible obstacles and successes. In order to gather this information, five case studies are presented and illustrated with a questionnaire that was carried out with the cities, researchers and key innovators. Results show that DWC´s co-creation process succeeded in incorporating end-users’ needs into the development of the digital solutions created, giving them a constructive role in the value-creation process, and bringing together different stakeholders from public and private sectors as well as researchers. It was also highlighted that the process and its format need to be adapted to each specific context and a culture of co-creation needs first to be established. The co-creation process and the DWC CoPs also raise governance questions and create new partnerships.

## Introduction: urban water management in Europe under changing environments ^1^

The EU water sector is facing increasing issues exacerbated by mounting pressures, e.g. growing population, urbanization and climate change. Agricultural and industrial activities, as well as increased urbanization, lead to pollution, overexploitation and modification of water bodies. Furthermore, almost three quarters of Europeans live in urban areas, with an upward trend that is projected to reach 83.7% by 2050 ( UN, 2018). Population growth, concentrated in European cities, will be accompanied by an increase in demand for water both inside and outside urban spheres. The main drivers will be agricultural production as well as industrial (energy and manufacturing) and domestic water use in cities ( Water Europe, 2017). Water scarcity - broadly defined as the mismatch between the resource availability and its consumption - occurs due to the increase in water demand and a reduction in availability. This increasing demand is a serious problem in arid and semi-arid areas of Europe. Concern around the water sector is all the more widespread as a result of climate change and its impacts ( EEA, 2021). Social and environmental systems are put under pressure due to rising risks of floods, an increased frequency and intensity of extreme precipitation. Too little or too much water can have adverse effects on water quality. For example, seawater intrusion and increased salinity can be driven by overexploitation of aquifers, while extreme rainfall can wash agricultural pollutants into rivers and lakes ( EC, 2019). Both scarcity and abundance of water can have detrimental effects on water quality, and when water quality is threatened, it can also reduce the availability of water for human consumption and for ecosystems sustenance.

These issues have implications for the entire water sector and all the involved stakeholders, particularly in the urban context. Every year, billions of dollars are invested in water treatment and sanitation infrastructure. They aim to improve health protection, performance and public engagement. In this context, and given the environmental, social, economic and structural issues, the management of the water cycle and its associated planning, development and operation are changing. These changes represent various challenges for the entire sector and its players.

Sector-specific challenges arise in dealing with the macro-environmental changes mentioned above and relate to governance aspects ( EEA, 2018), such as ensuring good policy fit (policy and regulation standards and boundaries), overcoming the limits of rigid institutional architectures, specifically between and within EU Member States, addressing slow innovation rate as well as low awareness and engagement of users, and providing supervisory measures to face inappropriate applications leading to substantial levels of illegal harvesting or use. Another challenge concerns the aging and lacking infrastructure. Finally, users’ awareness and engagement are fundamental points ( Dige *et al.*, 2017; Silva Pinto *et al.*, 2018). These processes can be put in place by raising awareness on resource management and use, as well as the uninterrupted access to drinking water and its implications.

In order to face these challenges, digital technologies can help. However, the integrated multi-scale governance of the water sector involves a complex interaction of agents, including political, economic, social, academic actors, etc. Moreover, it is necessary to focus on solution integration and inclusive governance for successful adoption. At the same time, long-term implementation of utilities can often only be derived from a transparent approach to social communication and co-creation ( Stein *et al.*, 2022) as well as from enhanced participation of the various stakeholders. This process faces a low level of maturity of digital solutions concerning the aspects of standardization, interoperability, cybersecurity and governance. There is also a lack of hard evidence of the benefits provided by digital solutions at each level of the water value chain. Promising solutions often do not reach real applications due to the lack of social and technical preparation, and a mismatch with expectations and needs of end-users.

Within this context, the digital-water.city project (DWC) developed local technological solutions over three years for five European cities (Berlin, Copenhagen, Sofia, Milan and Paris). By gathering different actors through a transversal and participative project across Europe, DWC aimed to strengthen and maximize their collaboration. In order to achieve this, the project established multi-scale and multi-actor communities of practice (CoPs). Within these CoPs, a co-creation process was carried out throughout the project. This paper presents the process and raises the following questions: (1) What is the role and impact of the co-creation process within the CoPs of the DWC project? (2) How has the co-creation process contributed to the achievement of the objectives pursued? (3) What lessons can be learned to support the success of future co-creation activities in the field of water management? To answer these questions, five case studies are presented and a questionnaire was set up and answered by the cities, project leaders and innovators. The results are presented and discussed in this article.

## Setting the scene: conceptualization of value co-creation and communities of practice

### The concept of value co-creation

The concept of value co-creation was introduced in the early 2000s by Prahalad and Ramaswamy ( Leclercq *et al.*, 2016). A certain number of authors have participated in situating the theoretical and practical approximations of the concept related to their field of research and application (among others, Chen *et al.*, 2012; Grönroos, 2008; Ind & Coates, 2013; Vargo, 2008; Zwass, 2010). However, the term lacks a consensus regarding its conceptualization ( Leclercq *et al.*, 2016). In this sense, Torraco (2005) proposed to deconstruct the concept, while other recent publications emphasize the need to deepen knowledge on the construct of the term with regard to its conceptualization ( Chen *et al.*, 2012; Ind & Coates, 2013), components ( Ind & Coates, 2013), consequences ( Fuchs *et al.*, 2013; Thompson & Malaviya, 2013), drivers ( Nambisan & Baron, 2009), different underlying processes ( Vargo *et al.*, 2008), and measurement ( Coviello & Joseph, 2012; Payne *et al.*, 2008).

Based on the literature review of Leclercq, Hammedi and Poncin (2016) of 181 articles published, the concept was defined as "a joint process during which value is reciprocally created for each of the actors (individuals, organizations or networks). These actors engage in the process by interacting and exchanging their resources. Interactions take place on an engagement platform where each actor shares their own resources, integrates resources offered by other actors, and potentially develops new resources through a learning process” ( Leclercq *et al.*, 2016, p6). The key components are: the value created, the actors involved and the engagement platforms.

The concept has been used and applied in different areas. For example, understanding the mechanisms of co-creation of value has been perceived as a priority in fields such as marketing research ( Ostrom *et al.*, 2010; Ostrom *et al.*, 2015), brand image ( Hatch, 2012; Merz *et al.*, 2009; Tynan *et al.*, 2010), distribution ( Andreu *et al.*, 2010), innovation ( Füller & Matzler, 2007; Füller *et al.*, 2011; Spohrer & Maglio, 2008), online communities ( Schau *et al.*, 2009), services ( Vargo & Lusch, 2008), customer relations ( Roggeveen *et al.*, 2012), etc. In terms of cooperation between agents, the academic world has investigated how collaborations between companies and end-users could be set up, such as collaborations of companies with consumers, the development of new products ( Riggs & Von Hippel, 1994; Von Hippel & Katz, 2002), services ( Grönroos, 2012), etc. Along with the necessity to cooperate with the end-users of applications and products developed, the importance of integrating other stakeholders - such as suppliers, public institutions, collaborators - into the processes has been underlined ( Prahalad & Ramaswamy, 2004a). A number of authors plead for a "transition", moving from a transactional perspective towards one that is more collaborative ( Leclercq *et al.*, 2016). Moreover, the contributions on the need for an evolution of marketing also highlight how the process of co-creation of value is enabled through the exchange of both knowledge and skills ( Prahalad & Ramaswamy, 2004a; Vargo & Lusch, 2004).

Value co-creation is an important paradigm in which a common body of theoretical statements has been developed and applied to different contexts. Outcomes from previous research showed that marketing and service science play a major role; however, hundreds of papers also acknowledge the constructive role for the end-user in the process ( Galvagno & Dalli, 2014). The role of subjective and lived experience of customers in value co-creation is an emerging theme in research, to gain a better understanding of how customers live the experience and how this could improve the effectiveness of companies looking to integrate customers and their employees into their processes ( Galvagno & Dalli, 2014).

### The quadruple helix approach for co-creation

**Figure 1.**
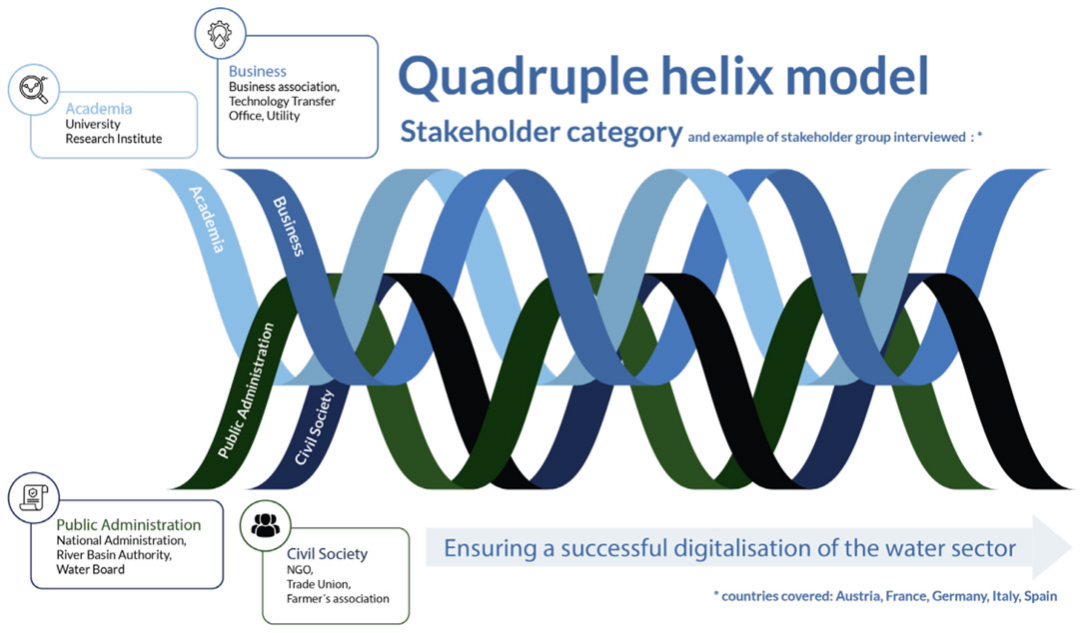
Anzaldúa *et al.* (2019) Quadruple helix model based on Carayannis & Campbelle (2009) and McIntosh & Gebrechorkos (2014).

In its Open Innovation 2.0 policy approach ( Curley & Salmelin, 2013), the European Union has adopted the quadruple helix model as a suggested approach for co-creation. According to this approach, adequate and operational solutions to meet challenges will emerge from the active involvement and collaboration between various key agents mentioned above: universities, businesses, civil society and public administration. The model showcases the fields of opportunities in which the actors are represented by “helices” where agents get involved together in order to ensure a reasonable digital transition. By acting in the areas of opportunity, the different stakeholder groups can influence the digital contribution in the water sector, highlighting the benefits and mitigating the risks and the barriers, and generating new possibilities, solutions and opportunities. The quadruple helix model is shown in Figure 1.

As explained by Anzaldúa *et al.* (2019), in terms of key stakeholders:

- The **Public Administration** plays a central role. They set the regulatory frameworks that the business sector needs to follow and respect; they facilitate control, compliance of solutions, standardization and prevention (for example for cyberattacks, illegal use of data, reasonable regulations of the private sector, etc.). Adequate public policies clarify responsibilities and obligations for the digital solutions installation and ensure positive social and environmental impacts. The administration also ensures the co-financing of training programs concerning the sector and its challenges.
- The **academic community** represents the other series of key agents. Researchers contribute to the modeling of applications (data, semantics, critical perspective, highlighting limitations, etc.). Advances in academic research play an important role in the process of raising awareness about digital technology and its utility for water management and treatment, as well as in advancing data democratization and information accessibility.
- The **economic and business sector** is considered another group of key actors. Companies are getting involved in public-private partnerships and generating investment in infrastructure and technologies. They have to take into account the current requirements of data protection laws and ensure that investments respect public regulations framework, remain reasonable by seeking a balanced and responsible deployment of digital solutions. They actively collaborate with academic researchers and can help to test new services. They have to adequately adapt their business models regarding the indications of the other actors' needs, concerns and expectations.
- **Civil society** is fundamental actor. It allows the public authorities and the private sector to assume their responsibilities for the decisions implemented, as well as their consequences. It is involved in training, engagement procedures and awareness-raising actions. This can be in regard to a good understanding of the supply and sanitation services and their associated costs, and help highlight their perspectives, needs and point of views.

This categorization of stakeholders into different groups follows the typology of McIntosh & Gebrechorkos (2014); the classification refers to the roles they play, specifically for the digitalization of the water sector. In the case of DWC, collaboration was facilitated between different actors, such as academics, companies, public administration and end-users, with the objective to develop and demonstrate digital solutions. As mentioned in the co-creation literature, there is a necessity to cooperate with the end-users of developed products, and integrate other stakeholders into the processes, such as public institutions and collaborators.

### The use of communities of practice for the adoption of the quadruple helix approach

Although the idea behind communities of practice has existed for much longer, the term “community of practice” was conceptualized and defined in the late 1980s ( Lave & Wenger, 1991). In its original conceptualization as proposed by the authors, communities of practice (CoPs) are formed by people engaging together in a process of collective learning.

CoPs have been conceptualized and practiced in many sectors and places. By creating a space for mutual learning and cooperation, these communities bring together groups of people who have a common concern or interest in a subject ( Wenger, 2010). In their formal definition, CoPs are “groups of people or organizations who share a concern or a passion for something they do and learn how to do it better as they interact regularly” ( Wenger-Trayner, 2015). Communities of practice can be seen as a social learning system ( Wenger, 2010). Learning in the interactions produces a social system where practice reflects one's own commitment to the situation. The dimension of identity enters into this process, where identification or dis-identification with and within the community takes place. The contributions of psychology showed how having the feeling of belonging to a group contributes to the constitution of identity itself. Theories of social identity and identification, from their foundations in social psychology - in this case applied to organizational psychology - demonstrated that people's self-identity concepts are partly constructed from the groups or collectives to which they think they belong ( Lee *et al.*, 2015; Tajfel, 1978; Tajfel & Turner, 1985). An essential aspect of the CoPs is thus the **community** itself. Several groups of people choose to engage in common activities and meetings, sharing information and developing skills and solutions together, making them members of a community. The process of interrelations and knowledge exchange allows them to learn together, and to understand the position and concerns of each member. Learning can be the reason the community comes together, or it can be the result of the members’ interactions.

In order to be considered a community of practice, certain characteristics are fundamental: interactions of CoP members with each other in formal and informal settings, knowledge sharing between them, collaboration to create new knowledge and the fact that CoP groups can promote the development of a shared identity between members ( Li *et al.*, 2009), even if these characteristics are not systematically present in the CoPs. They are also part of larger social systems involving other communities, structures, groups, etc., constituting the social world in which people and groups learn through a multiplicity of practices.

The concept of communities of practice has influenced, at a theoretical and practical level, a wide variety of academic fields, as well as the education sector, governments, public health, civil sector, private sector, etc. In the private sector, it is above all a space for peer-to-peer learning among practitioners in order to develop skills and capacities, and carry out different missions. In general, the communities of practice offer a promising organizational form to enable the development and implementation of socially beneficial innovation ( López-Gunn *et al.*, 2021) while involving the range of stakeholders and sectors considered by the quadruple helix approach.

## Communities of practice to support digitization in urban water management

As explained above, the water sector represents a complex network of issues and actors. This paper describes the activities conducted within the frame of the H2020 digital-water.city project (DWC) to bring together key actors and agents and make them work together in a framework, helping and assessing them to collaborate and provide relevant solutions towards the digitization of urban water management in European cities.

DWC has adopted the use of communities of practice as a central tool to help face these issues through collaboration between different actors and overcome barriers. The aim was to achieve an interdisciplinary and transdisciplinary approach through the integration of outcomes from several disciplines, and from academic, non-academic and non-formalized knowledge. This means that CoPs participated in both the formulation of objectives and the expected results.

The definition of communities of practice (CoP) is used in the project as: *a group of large and diverse actors who may be relevant to solving a problem and may be available to share and join experiences, skills, ideas, resources, actions to go further by adopting collective principles and shared societal challenges*. The CoPs create a dynamic learning process and a living collective body which, in the case of DWC, is meant to evolve by building trust between partners and achieving goals together : developing digital solutions adapted to the needs, and facilitating their local adoption. They are built of public, academic and private stakeholders from different backgrounds, fields and expertise, with the common goal of contributing to the development of solutions for water and sanitation infrastructure challenges.

The integrated multi-scale management of the water sector and its complex interaction of political, social, academic and economic agents is a challenge that DWC had to face, considering the fact that each agent has specific needs, expectations and concerns.

**Figure 2.**
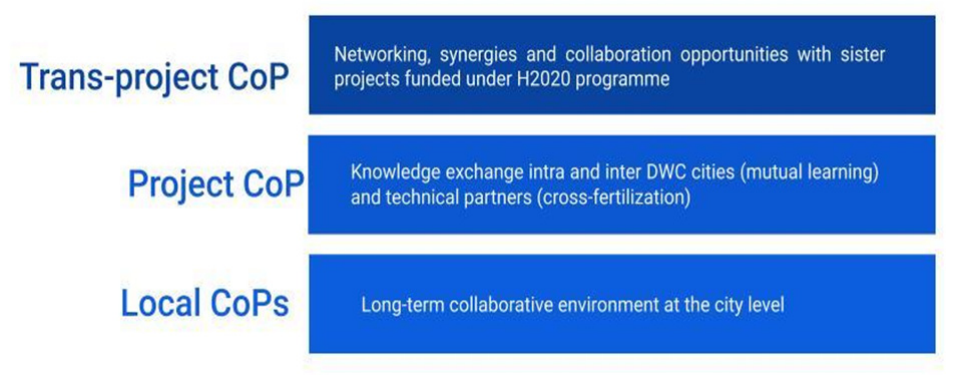
Multi-level Communities of Practice of DWC.

In order to implement the participatory process of learning through exchange and to accommodate this multiplicity of stakeholders, DWC's distinctive and unique feature was the establishment of different CoPs at three levels, as shown in Figure 2 below.

- Local CoPs (level 1), which have been operationalized in four European cities (i.e. Berlin, Copenhagen, Milan and Paris), mainly focus on the solutions within their local contexts. Local CoPs have aimed to: create a long-term collaborative environment at the city level during the project; increase knowledge exchange among local actors; support the integration of stakeholder knowledge and expectations into the development of solutions; and co-build trust of external stakeholders in the future use of solutions. Local CoPs have provided direct support for testing and implementing solutions in practical contexts, building trust in the usefulness and relevance of what was developed, and receiving specific user requirements and needs identified throughout the later stages of development. This is all the more relevant as innovations may tend to focus on technical aspects and partially neglect to take into account problems or difficulties related to the daily routines of end users. In addition, each of the cities faced different contextual challenges, and solutions and activities needed to be adapted by collaborating with local key actors and populations.
- Project CoP (level 2) provided a useful mechanism to facilitate knowledge exchange between DWC cities (mutual learning) and between DWC cities and technical partners (cross-fertilization). In terms of mutual learning, it was an opportunity for the cities to exchange their experiences regarding the new solutions, while comparing them to current solutions used to address similar problems. The objective of these activities was to identify drivers of and barriers to their adoption. Key aspects to be addressed include reflecting on the results of the local demonstration: what worked well (key factors), what were the implementation issues (main barriers and drawbacks), what could have been done differently, and what would be considered for reproduction in another setting? In terms of cross-fertilization, this CoP provided a space for discussion around cross-cutting topics addressed by the project (such as cyber-security, interoperability, digital governance, etc.), where technical partners and DWC cities met and shared doubts and experiences.
- Trans-project CoP (level 3) focused on networking and grouping activities with other related projects and actions. This CoP benefited from the DigitalWater2020 initiative, which partnered five sister projects funded under the H2020 program (i.e., SCOREwater, Fiware4Water, Aqua3S, Naiades, and DWC) to identify synergies and collaboration opportunities in several aspects, i.e., data models and ontology, sensors, markets, and communication.

These CoPs have been active for three years and involved the participation of hundreds of people representing different organizations (although often participating on the basis of their own expertise and knowledge). After this large effort in terms of organization of activities and devoted resources, this paper intends to answer three questions:

(1) What is the role and impact of the co-creation process within the CoPs of the DWC project?

(2) How has the co-creation process contributed to the achievement of the objectives pursued?

(3) What lessons have been learned so far?

In order to answer these questions, a mixed methodology was applied, with a survey and multiple case studies. This mixed approach offers advantages for researchers in addressing the complexity of these research problems and issues ( Plano Clark *et al.*, 2018). Combining methods allows combining external and objective observations and more subjective and socially constructed aspects.

## Methodology for assessing the impact of co-creation and CoPs

The approach has focused on identifying the impact of the activity of the Local CoPs in the five DWC pilot cities for supporting the development and potential adoption of innovative digital water solutions. A mixed methodology has been applied building on multiple case studies, surveys, interviews and a workshop mainly involving the pilot and city leaders. The decision to work with the pilot leaders was made because these actors have a complete view of the full process undertaken and can help document the main achievements and shortcomings, whereas other stakeholders generally have only partly participated in some of the activities organized by the local CoPs. In the case of each pilot city, either public or private entities were involved, which share a strong interest in developing and adopting innovations for improved digital management of their assets and infrastructure.

### DWC local COPs in action

***Initial characterization of the local COPs*.** The DWC project and the development of local digital solutions were active in five European urban and peri-urban areas representing 30 million European citizens. Due to the context and key challenges varying across the five participating cities, the format of the Local CoPs (CoP level1) was organized in different ways throughout the project, as shown in the Table 1, Table 2, Table 3, Table 4 and Table 5.

**Table 1.** Berlin Local CoPs (CoP level1).

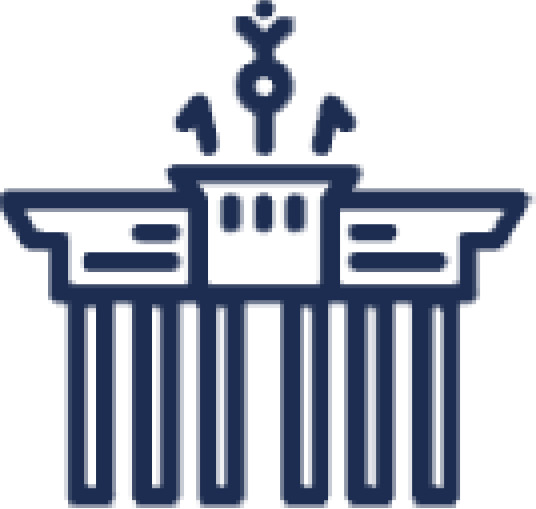 Led by **Berliner Wasserbetriebe (BWB)** **Key Stakeholders**: Berlin Water Works (BWB), Berlin Senate, Berlin Waste Management (BSR),State Office for Health and Social Affairs Berlin (LaGeSo), German Federal Environmental Agency (UBA), KWB, Berlin Partner
**Goal and ambition:** The objectives for Berlin were to improve the performance of the sewer and drinking water infrastructure and foster public involvement in urban water management. Several innovations will reduce the environmental impacts of the sewer network. The work focused on illicit connections, combined sewer overflows and optimizing the maintenance and planning of water wells. Water management was considered in the entirety of the city, allowing local stakeholders to benefit from the solutions. The expectations and requirements of local stakeholders were included in the development of the digital solutions.
**Digital water innovations supported:** The augmented reality App "Grundwasser sichtbar machen" developed for Berlin aimed to visualize geology and groundwater and highlight their importance as a drinking water supply and to make this invisible part of the water cycle visible. The application will be used for many communication objectives (education, tourism), with the overall goal of raising knowledge about the origins of drinking water and communicating the relevance of groundwater for municipal water supply. As a result, the application addresses three key issues: 1) Where does the drinking water come from? 2) How does water enter the wells? 3) How is water cleaned during the soil-aquifer transition?
**How the CoP worked. Formal decision making and/or only information. What was discussed:** The CoP worked through meetings and workshops (using a brainstorming/exchange approach), including a presentation of the project (structure, key goals and ambitions), the digital solutions being developed and implemented in Berlin, and through participatory workshops. The methods and results obtained in the meetings were presented to the other DWC cities in the project's general assembly.
**Key challenges related to local digital water governance aspects**: The urban water cycle is partially closed and challenged intensively by competing uses and pressures such as drinking water production, discharges of stormwater and treated wastewater; combined sewer overflow (CSO) and recreational purposes. The increasingly digital agenda of Berlin leaves room for the application of ICT technologies in water management. However, the growing amount of digitalization programs and legislatives schemes has not yet been quite impactful in the water sector. Local policies for the digital sphere take the form of action plans and strategies rather than legally obligatory regulations. The interaction between digital and water policies is not yet well established. There is currently little cross-sectoral focus on the use of digital technologies on water management and environmental education. Relevant networks that foster collaboration and cooperation in digital water still need to be developed.

**Table 2.** Paris Local CoPs (CoP level1).

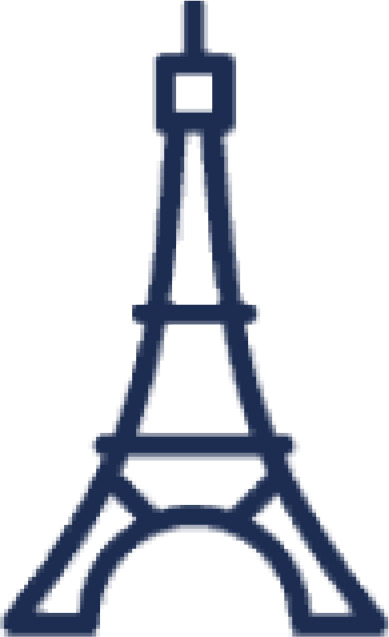 Led by **SIAAP** **Key Stakeholders**: SIAAP, INRAE, The City of Paris; The health and environmental authorities; The Seine-Normandy water agency; The Direction of the services for water and sewerage of the Val de Marne département; The Direction of water and sewerage of the Seine Saint Denis département; The Direction of water of the Haut de Seine département; The Syndicat Marne Vive.
**Goal and ambition:** One of the goals of the project was to involve the future stakeholders (citizens and bathing sites managers) in the development of two specific digital solutions, a “public” app and an “expert” app.
**Digital water innovations supported:** The innovations supported were the development of two specific digital solutions: an “Expert” application destined to bathing site managers that will regroup all the information needed to decide whether to open or close a bathing site and a “Public” application destined to inform the citizens of the status of the bathing site of their choice.
**How the CoP worked. Formal decision making and/or only information. What was discussed:** One of the original aspects of the CoP was the fully participatory nature of both applications, which brought together all the actors working on the Paris bathing situation, as well as representatives of other cities interested in opening a bathing site. The CoP worked with regular meetings and workshops. In the Paris experiment, the focus group method (focused discussion where the interaction between the participants creates the data) was used to assess the expectations and apprehensions of the "general public" with regard to the return of bathing in urban streams. This method guided the development of the digital application intended for future users of bathing sites.
**Key challenges related to local digital water governance aspects**: In a framework of supporting the organization of the next 2024 Olympic Games, a working group involving a large number of stakeholders and actors (including SIAAP) collaborated to improve water quality status and monitoring in river Seine. SIAAP is an active member of this “bathing task force” which has been established in order to reach the goal of sufficient water quality for bathing. One of the challenges was to involve the citizens and the future bathing site managers in the development of the digital solution for the Parisian region project. The Paris case also faced several political challenges regarding bathing site implementation and the overall question of bathing.

**Table 3.** Milan Local CoPs (CoP level1).

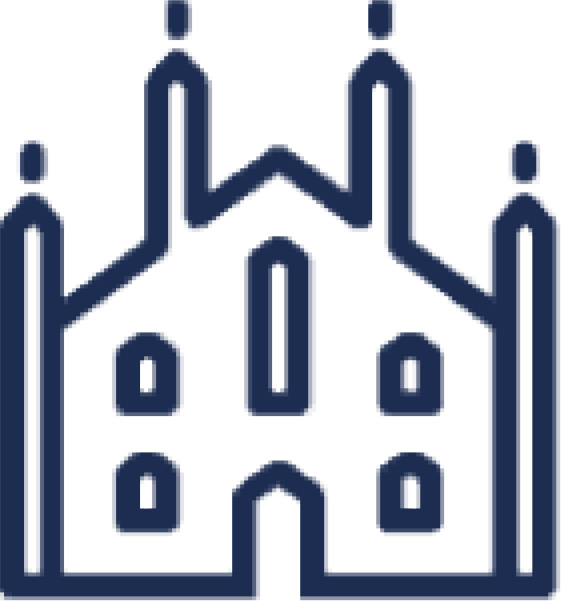 Led by **CAP Holding, Università di Milano, Università Politecnica delle Marche** **Key Stakeholders**: CAP Holding, Università di Milano, Università Politecnica delle Marche, three large farmers’ associations and public bodies (COLDIRETTI, Confagricoltura, ETC-Villoresi), Milan municipality.
**Goal and ambition:** Deployment of a reused wastewater network for agricultural irrigation in the Milan area, and sanitary concerns about water quality.
**Digital water innovations supported:** A match-making tool which links water demand for irrigation and safe water availability, the active unmanned aerial vehicle for analysis of irrigation efficiency, the serious game on the water reuse-carbon-energy-food-climate nexus, and the Early Warning System for safe reuse of treated wastewater for agricultural irrigation. The serious game developed by the Marche Polytechnic University as a tool for communicating and understanding the process of irrigation reuse of purified wastewater and the link between water, energy, food and climate.
**How the CoP worked. Formal decision making and/or only information. What was discussed:** The CoP worked with regular online and face-to-face meetings, including presentations of the development progress of the solutions, updates on implementation, demo, testing activities and hands-on play sessions. Feedback from the stakeholders was collected using Slido software, adapting to the relevance of the solutions for the Milan context. The specific testing activities were for the development of an integrated wastewater management and reuse system - with a particular focus on risk management aspects, the Early Warning System, digital tools to support Early Warning at the Peschiera Borromeo wastewater treatment plant, match-making tool and next irrigation test campaigns with reused water, as well as the serious game on Water-Energy-Carbon-Climatic nexus.
**Key challenges related to local digital water governance aspects**: The challenges were: the preferred time-period to receive information about availability of re-used water for its use in irrigation; importance of the consideration of the carbon footprint of water used for irrigation; relevance of solutions for the improvement of agricultural productivity and sustainability; and initial interest from stakeholders to the presented solutions.

**Table 4.** Copenhagen Local CoPs (CoP level1).

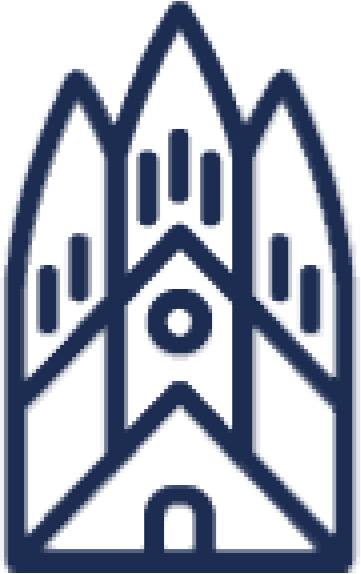 Led by **BIOFOS** **Key Stakeholders**: Ongoing operational group for integrated water management involving BIOFOS and other water utilities in the Copenhagen greater region
**Goal and ambition:** The objectives for Copenhagen were to reduce environmental impacts and flooding and improve the stability and biological capacity of the wastewater treatment plant (WWTP). The project aims to reduce environmental impacts and flooding through flow forecasting and real-time control between the sewer network and a wastewater treatment plant.
**Digital water innovations supported:** The web-based tool SAMDUS is an interoperable visualization platform that provides data and analytics to all stakeholders responsible for the integrated management of sewer networks and wastewater treatment plants (WWTPs) in an urban area. The platform enables the sharing and visualization of data from a series of sensors, models and decision support systems. It integrates the total system dynamics and facilitates real-time decision-making across all utilities and entities, increasing preparedness for high-flow events; Another solution is the sewer flow forecast toolbox, which is a machine-learning (ML) based tool for forecasting flow in the sewer network and inflow to the wastewater treatment plant (WWTP), with forecast lead time of up to 48 hours. The tool is based on a combination of (1) real-time water level and flow sensor data from the sewer system, (2) rain gauge data, (3) weather radar observations and nowcasts, and (4) weather forecasts from numerical weather prediction models. The solution provides an accurate flow modeling forecast and horizon to support the integrated management of the sewer network and WWTP. A third solution is a Decision Support System (DSS), which is an innovative tool for the sustainable operation of the integrated sewer network and wastewater treatment plant (WWTP). It is based on a series of level and flow sensors within the sewer network, WWTP operation data and accurate flow forecast at the inflow of the WWTP. The goal of the DSS is to optimize treatment capacities, pumping strategies and in-sewer flow allocation to make the best use of existing infrastructure for stormwater handling.
**How the CoP worked. Formal decision making and/or only information. What was discussed:** The Copenhagen case reunited a group of stakeholders to meet regularly and share expectations which can be used for the product development. The CoP process worked through an ongoing operational group for integrated water management involving BIOFOS and other water utilities in greater Copenhagen. This group includes the key actors to support the development of the digital solutions being developed and tested in Copenhagen. Feedback from other water utilities was very relevant to the development of the “Web platform for integrated sewer and wastewater treatment plant control”, which is also directly related to other two DWC solutions (i.e. sewer flow forecast toolbox, and the interoperable DSS for stormwater management). A presentation of DWC Copenhagen and the solutions, and several workshops were organized where information was collected in a systematic way, bringing together different views and expectations for the solutions and then further exploring the comprehension of the overall context and the specific attributes which may be desirable for the key stakeholders.
**Key challenges related to local digital water governance aspects**: BIOFOS takes care of wastewater treatment in Copenhagen, while HOFOR supplies 1.1 million customers with drinking water, district heating and wastewater management. Current challenges are: real-time control of stormwater being hampered by lack of accuracy of the wastewater treatment plant’s flow forecast and lack of interoperability between the data management systems of BIOFOS and HOFOR.

**Table 5.** Sofia Local CoPs (CoP level1).

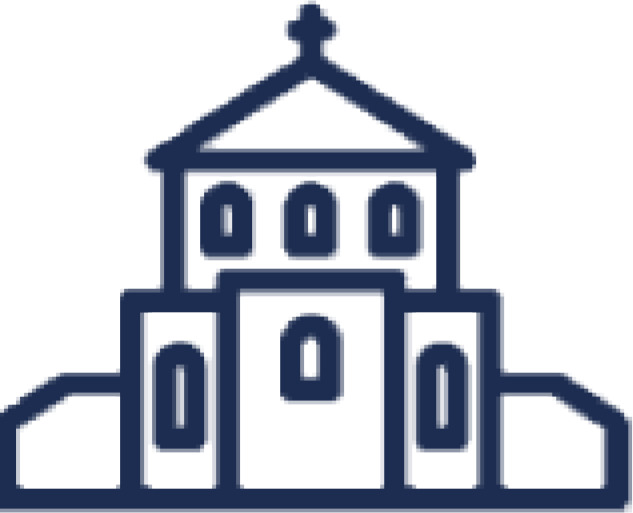 Led by **Sofiyska Voda AD** **Key Stakeholders**: Veolia group, Clean & Circle Competence Center, Municipalities of Sofia region, Bulgarian water utilities, scientific community in Sofia (University of Architecture, Civil Engineering and Geodesy in Sofia and others), commercial companies carrying out digital installations.
**Goal and ambition:** The objective in Sofia was to create awareness in target audiences about the project objectives and progress.
**Digital water innovations supported:** The solution developed was the low-cost temperature sensors for real-time combined sewer overflow (CSO) and flood monitoring, which is based on the deployment of a network of innovative low-cost temperature sensors to estimate emissions from combined sewer overflows (CSO) across a large number of points in a sewer system. CSO events and their duration can be detected by shifts in temperature within the sewers, thanks to the temperature difference between the air phase and stormwater discharge.
**How the CoP worked. Formal decision making and/or only information. What was discussed:** A series of information and dissemination activities was organized about the DWC project, both among employees and citizens. The DWC project was also presented to the young audience – high school students from the Sofia High School of Construction, Architecture and Geodesy “HristoBotev”, and Open "Sewer" classes in front of the same high school.
**Key challenges related to local digital water governance aspects**: One of the challenges was to increase sewer maintenance efficiency in order to reduce blockages and flooded properties, as well as increase customer satisfaction. Therefore, the objectives for Sofia are to optimize future investment through better data availability on combined sewer overflows and reduce operational costs for sewer cleaning and inspections.

***Differences and commonalities*.** As an initial step, the DWC cities participated in a World Café exercise organized in the initial months, which allowed for the elucidation of commonalities and differences in their approaches and expectations for the local CoPs:

Based on the discussions, a number of common points among several DWC cities were identified:

1. Cities are generally very interested in **exchanging experiences** with other cities, e.g. learning how other cities are implementing the solutions or which kind of barriers or limitations have been identified. Thus, **mutual learning** was clearly identified as the key aim for the project CoP.
2. Some of the cities envisaged **collaborations with ongoing projects** or initiatives at the local scale. These kinds of networking activities should provide opportunities for improved adoption and/or dissemination of the expected results, and the local CoPs were recognized as valuable vehicles to support these kinds of interactions.
3. A crucial aspect to be decided was **when to engage with the relevant stakeholders** (e.g. from the beginning, once initial results are available, when solutions are sufficiently tested, at the end of the project for communication and dissemination purposes, etc.). Every pilot city made its own decision on this aspect depending on its specific context.
4. One of the key reasons identified for involving stakeholders into the project activities was **building trust** through the stakeholder engagement in the local CoPs. Related to this point, it was argued that the involvement of operational teams may be particularly remarkable in order to improve the usability of some solutions.
5. **Data exchange** was considered a significant challenge for many of the cities. There is a need to better understand which data exchange is needed, and which data can be exchanged, i.e. open data/critical data. It was identified as a significant potential to help unravel these aspects through the local CoPs.

In addition to the common points, a number of differences were recognized:

1. There are different needs regarding stakeholder **involvement**, i.e. ranging from a strong interest by stakeholders to cooperate in the co-development of some solutions to cases where the need of stakeholder involvement is very limited. It was agreed that this factor needs to be strongly taken into account for the design of the activities of the Local CoPs.

2. The required **stakeholder involvement** can be related to different stages of development, namely:

- contribution for the specification of **technical characteristics**
- consideration of **expectations of end-users** throughout the solution design and development
- **convincing** end-users and stakeholders **about the benefits** from the implementation
- increase of **communication and dissemination** of the results

3. There are differences in **problem awareness** of public authorities. For some DSs, there is a clear willingness of public authorities to cooperate to reduce existing problems (e.g. bathing quality) whereas it is not fully clear whether public authorities have a real interest in strongly contributing to sort out other problems (e.g. detection of illegal sewer connections). As for the latter case, there was an acknowledgement of the need to make an effort to involve authorities so as to motivate them to work on the topic.

4. The discussions showed that in the different cities, **digitalization has been integrated quite differently.** Often, technical aspects of implementing new technologies and making them work prevail over ICT governance and policy aspects.

All these points have been considered for the identification of the impact delivered by the local CoPs.

### Reflecting on the process of co-creation

The aim of reflecting on the general process of co-creation was to get standardized and comprehensive feedback building on the direct feedback collected from the cities and project leaders.

As a first step, a survey was circulated to the pilot leaders to document the scope of co-creation activities within the DWC CoPs. In most of the cases, a follow-up interview was conducted to complete the information and discuss potential improvements in the participatory work plan that could better support co-creation within the CoPs. These were semi-structured interviews following a predefined questionnaire, divided into six parts: Objectives (co-development of solutions to overcome barriers from innovation to practice; Collect user requirements; Business development and contribution to value; Collaboration in co-creation); Process; Unexpected results. For each category, the details of the questions are presented in the Table 6 below.

**Table 6.** Questions to collect feedback on the co-creation process of the DWC Communities of Practice.

Objectives	Questions
Co-development of solutions to overcome barriers from innovation to practice	*Has co-creation been achieved? If yes, at what level (project activities/between cities/within the city)?*
	*Has this co-creation process contributed in any way to improving the development of digital innovations?*
	*Were the set goals achieved?*
Collect user requirements	*Have the needs of all users been collected? Yes/No. If yes, in what way? If not, why?*
	*Who were the actors most involved in this co-creation? (Please explain)*
Business development and contribution to value	*How does co-creation contribute beyond what the economy offers? Possible answers: specific solutions to* * a specific problem based on the development of existing generic solutions / Solutions more adapted to the * *operating process / New solutions not yet considered by the market / Other*
Collaboration in co- creation	*Does co-creation in CoPs offer new opportunities for cooperation with other stakeholders? Possible answers: * *Definitely yes/somewhat/not really (You can find space below to expand on your answer if you wish)*
	*In case the co-creation did not achieve the objectives, could you explain why?*
	*If the expected co-creation is not produced, what do you think are the main causes or barriers? Possible answers: * *Technologies are not developed/ Lack of stakeholders/ The design of the participatory process is not adequate/* * Lack of communication/adequate dialogue/ Online communication barriers/ Other*
Process	*Was the co-creation process (format, activities, actors, etc.) implemented adequate to achieve the objectives? * *Yes/No (You can find space below to expand on your answer if you wish)*
	*How did this co-creation process go? Possible answers: During CoPs/ Individual interaction/ Information sharing/ * *Other*
	*How could this process be improved?*
	*Why would co-creation be considered fundamental/necessary?*
Unexpected results	*What unexpected results did co-creation bring? (You can find space below to expand on your answer if you wish)*

**Figure 3.**
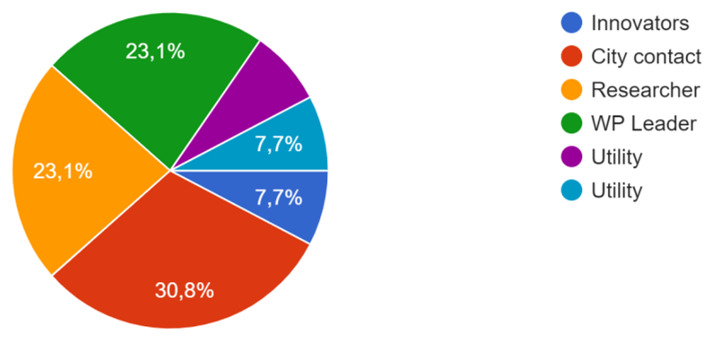
Participants of the survey.

The survey, produced as a questionnaire, was applied to pilot leaders, to consortium researchers and stakeholders involved in the local CoPs, as well as to some innovators (see Figure 3). 

The answers to the survey were collected during an online meeting, creating an informal space from a broader dialogue and debate on co-creation within DWC, thus allowing the actors to discuss and share their experiences at the final stage of the project.

## Outcomes

The participatory approach of the project was implemented within the Communities of Practice, at different levels, as presented previously. This section presents the results of the survey for each group of questions and incorporates the reflections about co-creation within local CoPs. The detailed answers to the survey can be found in Table 7.

**Table 7.** Detailed results of survey.

Objectives	Questions	Answers
Co-development of solutions to overcome barriers from innovation to practice	*Has co-creation been* * achieved? If yes, at what* * level (project activities/* *between cities/within the* * city)?*	Yes, at the city level
		Yes, project activities
		Yes, project activities and exchanges between cities
		Project activities
		Local level
		Yes, on project activities
		Yes, in the city and between Paris and Berlin
		Not at the local level, at the project level
		Co-creation at local/city level as well as at project level has been achieved
		From our point of view (with a lack of visibility on the co-creation process of applications), co-creation has been particularly effective at the city level where the opinions gathered during communities of practice and focus groups have been incorporated into the development of applications.
	*Has this co-creation* * process contributed in* * any way to improving the* * development of digital* * innovations?*	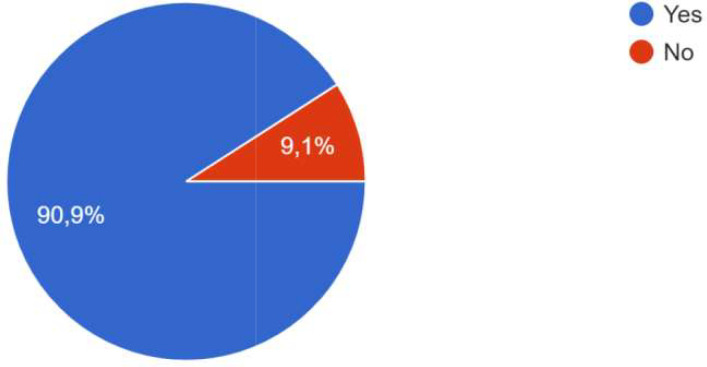
	*Did the goals set were * *achieved?*	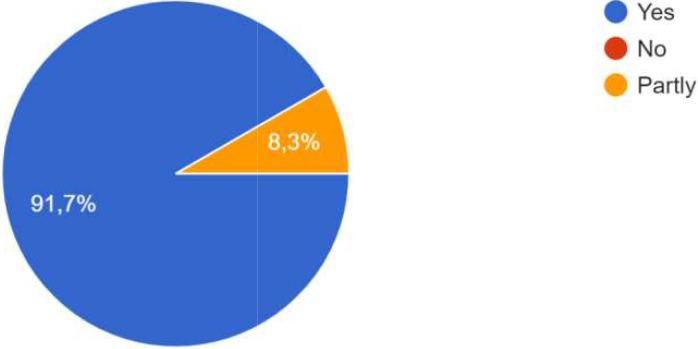
Collect user requirements	*Have the needs of all users * *been collected? Yes/No. If* * yes, in what way? If not,* * why?*	Yes, through COP and involvement in product development.
		Yes, during multiple meeting organized (once a month) and email exchange with tables to fill in
		Yes, we arranged meetings with utilities to know their needs
		Difficult to say in our developments. Probably not the needs of all users, but quite a few needs were collected and addressed.
		Depends on cities
		Yes. but not all is implemented.
		Yes. But for the public app, we had to take into account the digital divide, the fact that many water users are not app users. So app users are a smaller population than what we initially thought
		I think so.
		Requirements for digital solutions, potential features have been collected during workshops and CoP actions.
		It would be illusory to try to represent the views of all future users of the applications - this is also one of the points that the project has helped us to understand. Enrolment in the co-creation process can only involve motivated individuals/ organisations (e.g. swimmers who already practice open water bathing or residents living close to future bathing sites who feel concerned by the issue and/or for whom the question of legitimacy to express themselves on the topic is less of a barrier).
	*Who were the actors* * most involved in this co-* *creation? (Please explain)*	Corresponding authorities.
		Stakeholders and the public.
		ICRA, BWB and Sofiyskavoda.
		Researchers & operators.
		BWB, P4uw, SIAPP, Amsterdam.
		Researchers, WWTPs, software developers.
		City leader + innovators.
		Innovator and BIOFOS as a user... Stakeholders partly via 2 workshops. BIOFOS on a regular basis.
		Wastewater managers and future bathing site managers
		Intra project partners.
		Utility and city representatives in local CoP; technology providers for digital solutions discussed
		It is more relevant and easier to answer this question from the example of the communities of practice because the focus groups only brought people together once. All our attempts to reproduce the experience failed, which testifies to the difficulty of maintaining the involvement of people expressing themselves in an individual capacity in a process of co-creation of an application - all the more so as the participants in these focus groups were systematically critical of the temporality of this co-creation process: how can we build an application aimed at providing information on bathing sites when these do not exist? The participants in the communities of practice were more likely to maintain their participation over the months. It can be noted, however, that the most consistent participants were the institutions that were best informed and "motivated" by bathing, while the representatives of municipalities that were still hesitant about their desire to create sites participated in a fluctuating way (absence from meetings and lack of intervention during them).
Business development and contribution to value	*How does co-creation* * contribute beyond what* * the economy offers? * *Possible answers: specific* * solutions to a specific* * problem based on the* * development of existing* * generic solutions/* *Solutions more adapted* * to the operating process/* *New solutions not yet* * considered by the market/* *Other*	Specific solutions to a specific problem based on the development of existing generic solutions/Solutions more adapted to the user needs.
		The idea was to use a co-creation process so that the tools developed correctly match the demand of the stakeholders and the public. The stakeholders contributed to building the tools by specifying the content. We believe that if they participated in the development process, they would be more willing to use the tool afterwards: they would have trust in it!
		Solutions more adapted to the operating process.
		Better feedback to new solutions - reducing failure rate of products.
		Solutions better adapted to the operating process.
		Specific solutions to a specific problem based on the development of existing generic solutions/Solutions more adapted to the operation process.
		Specific solutions to specific needs expressed by users: alerts for the expert app, additional information for the public app.
		Solutions more adapted to the operating process.
		Agile development of digital solution made, solutions more adapted to the operating process.
		I don't know.
Collaboration in co- creation	*Does co-creation in CoPs* * offer new opportunities * *for cooperation with other * *stakeholders? Possible* * answers: definitely yes/* *somewhat/not really (You * *can find space below to* * expand on your answer if * *you wish)*	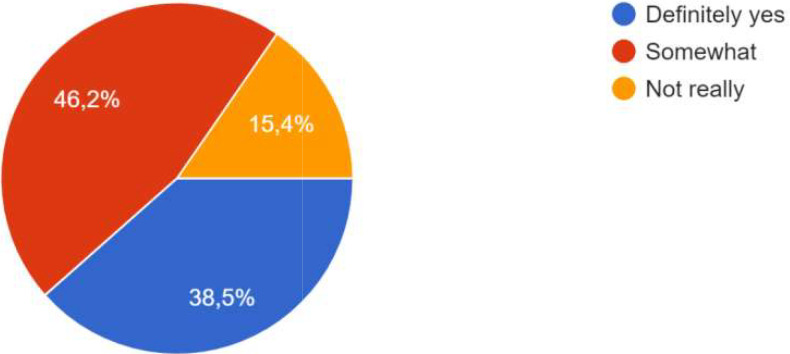
	*In case the co-creation did* * not achieve the objectives,* * could you explain why?*	The co-creation process is quite time intensive. There was no established culture of co-creating. Thus, the learning curve was steep for all actors involved.
		Lack of time for the procedure
		Hard to involve external partners in new processes without funding
	*If the expected co-* *creation is not produced, * *what do you think* * are the main causes * *or barriers? Possible * *answers: Technologies * *are not developed/Lack * *of stakeholders/The* * design of the participatory* * process is not adequate/* *Lack of communication/* *adequate dialogue/Online* * communication barriers/* * Other*	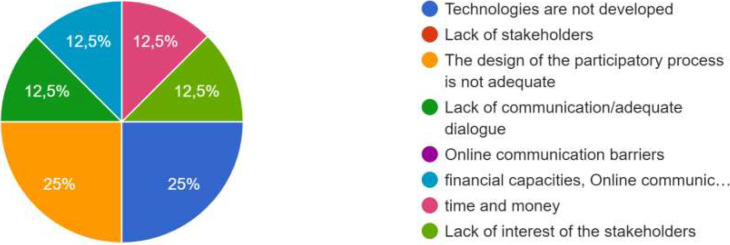
Process	*Was the co-creation* * process (format, activities,* * actors, etc.) implemented* * adequate to achieve the* * objectives? Yes/No (You* * can find space below to* * expand on your answer if * *you wish)*	Yes
		Yes! We wanted them to participate without blocking too much of their time, so we decided to fix one meeting per month and exchange information via emails.
		It was a starting point
		We had a great exchange webinar about illicit connections (ds9) as follow up action of the CoPs. Discussed different techniques and strategies to deal with illicit connections
		Yes.
		Yes.
		Yes. But can be done better.
		The co-creation process that was put in place took time to set up; it required prior preparation work to find the format most likely to attract the participants (work carried out by Sofia Housni). The institutional stakes were high in Paris with a diversity of actors and territories represented in the CoPs, with a divergent level of knowledge and involvement in the bathing issue. The key step that enabled the effective calibration of the CoPs was the preliminary individual interviews with the representatives of the invited institutions to explain the approach and find out about the institution's position on the subject. Another fundamental step was to agree on the mode of deliberation for collective decision-making. Digital tools were effectively used to this end to survey participants' opinions.
	*How did this co-creation* * process go? Possible * *answers: During COPs/* *Individual interaction/* * Information sharing/* * Other*	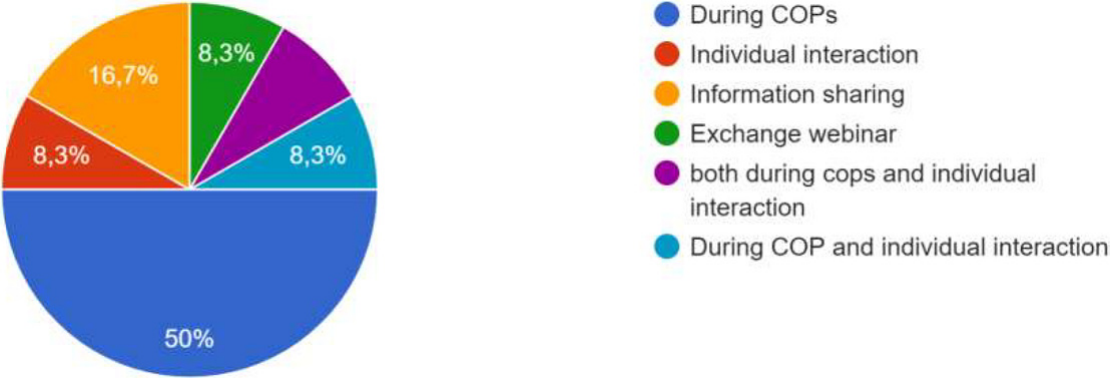
	*How could this process be * *improved?*	Align the co-creation process closer to the user's needs.
		We believe that it went really well considering the amount of people involved and we offered different options so that everybody had a say such as specific individual meetings outside of COPs meeting
		More physical meetings
		Design of the event, better highlight the needs and added value of the co-creation
		Smaller ambitions.
		More structured results from the CoPs
		Hard to do. Stakeholders in COP do not have capacity and budget for more involvement in the co creation
		An important lesson from the process of co-creation that we invite reflection on is that of the embeddedness of this practice in other wider practices and concerns. Sensitivity to this point must be kept in mind in order to maintain the involvement of the participants and make it effective (one must be ready to see the initial plan bifurcate by listening to the actors' concerns and constraints). In the case of bathing, participants needed to be informed of the implication of future site management beyond the sole issue of water quality. Their confidence and involvement in the co-creation process was maintained by listening and responding to these concerns.
	*Why would co-creation be* * considered fundamental/necessary?*	Because it establishes a joint learning culture.
		Involving the future users in the development of the tool allowed us to create something that perfectly answers their demands, and we were able to build trust in the tool so that they could use it with a lot of confidence
		For us, because we were able to know the specific needs of our partners
		Different sectors - different backgrounds and competencies
		To develop solutions people want to use.
		Because the assumptions made by developers about users' need are often biased
		It is a platform for sharing.
		The case of the development of the "general public" application in Paris demonstrates the importance of the co-creation process, which allows stakeholders to be heard beyond the commercial approach of assessing the application's ergonomics or the accuracy of the technical information on water quality. It has thus made it possible to identify the need to integrate awareness and prevention pages on the risks associated with swimming (linked to navigation, the presence of dangerous objects in the water).
Unexpected results	*What unexpected results* * did co-creation bring? (You* * can find space below to* * expand on your answer if* * you wish)*	Cooperation between the different actors (those who had a better understanding of the tools and the situation and those who didn't), cooperation with outside participants
		Communication
		Prioritizing. Only select those features that are relevant to the most end-users. One must be aware of benefits and performance. This was more important than the feature itself here in Copenhagen.
		New questions of governance.
		(see previous answer on the need for information beyond the sole water quality issue).

### Achievements

The co-creation has resulted in several achievements and successes. The majority of respondents indicate in particular that this co-creation process contributed to improving the development of digital solutions through different aspects.

•    Cooperation:

As explained by the participants, co-creation allowed more cooperation by bringing together different actors from public, private and research fields. It allows stakeholders to be, as said one participant, “heard beyond the commercial approach of assessing the application's ergonomics or the accuracy of the technical information”. Co-creation brought more communication and “cooperation between the different actors (those who had a better understanding of the tools and the situation and those who didn't)” and cooperation outside the participants’ project. Co-creation was fundamental because it made work “different sectors” from “different backgrounds and competencies”, and established between the actors of a project a “joint learning culture”. More generally on communities of practice, it was said that these mutual learning spaces offered new opportunities for cooperation with other stakeholders.

•    End-users needs:

One of the main achievements of co-creation is that the needs of users have been collected. One participant indicated that “probably not the needs of all users, but quite a few needs were collected and addressed” and another participant explained that through co-creation it has been able to make them understand that it is not possible to represent the viewpoints of all users of the solutions developed.

Including end-users’ feedback made it possible to “develop solutions people want to use”, and was necessary “because the assumptions made by developers about users' needs are often biased”, as mentioned by some participants. Co-creation provides the means to gain an in-depth understanding of the needs and requirements of the future users.

In the case of Berlin, users’ fear of information overflow was identified, as was the lack of staff and resources to maintain and operate the digital solutions after a pilot phase, as well as the trade-off between the required easy availability of data and the need of secure data to protect critical infrastructure. Some participants highlighted the high specificity of the solutions developed “to a specific problem based on the development of existing generic solutions” (for example some aspects were improved, and it is now possible to add new scenarios with few additional developments input). Through co-creation, this could lead to a reduction of a product’s failure rate, or to solutions that are better adapted to the needs of end-users.

In the case of Copenhagen, through co-creation, the identification of several key aspects has led to enhanced product development (for example, water utilities are either do not regularly use current systems for data sharing, or they limit their use to the analysis of rain events or for getting a quick overview over WWTP and sewer system performance). In terms of expected improvements, the utilities identified several specific attributes, such as scenarios calculation and evaluation, integrated graphs, an overview of different variables, etc. Moreover, participants recognized a number of expected benefits (e.g. increased cooperation, improved internal and external communication and savings in operation costs) and expected experience in the use of the system (e.g. easy data visualization and download).

In Milan, an important lesson learned is that, although these stakeholders provided highly valuable feedback, it was concluded that a greater involvement of end-users into this exercise can help to better tune-up and refine the tool.

In Sofia, co-creation focused on communication of results (i.e. how to regularly report on the collected data and generated information to the stakeholders) as well as on promoting the use and further adoption of the digital solutions and the information generated through their use.

In Paris, the end-users’ feedback has been incorporated into the applications. In the case of the Paris region, the discussions during the Focus Group went far beyond the question of the digital tool, and the reactions collected are valuable lessons concerning the general issue of bathing in Ile-de-France ( Rouillé-Kielo & Bouleau, 2021). The participants had the particularity of being interested and invested in the subject of the return to swimming and/or the deployment of digital information tools. These people could share their knowledge of the territory and/or the subject, even if they sometimes had to be reassured about their legitimacy to express themselves and to be listened to. Mutual acquaintance or commonalities between participants constituted strong enough foundations to allow a free flow of speech. Regarding the subject of bathing and risk, the discussions during the participatory process raised other questions and largely went beyond the framework of the development of digital applications and concerned the adaptation of facilities and communication to future bathing uses. Many participants insisted on the fact that the existence of different audiences should be anticipated at bathing sites, depending on the type of bathing site. These audiences will not have the same expectations in terms of equipment, information, opening hours, and should be taken into account to adapt the offer to a site or between several sites on the metropolitan territory. The question of conflicts of use on the banks and in the water was therefore a central element in the discussions. It concerned the question of the preservation of biodiversity which could be threatened by overcrowding. A final type of conflict mentioned during these discussions is between legitimate and non-legitimate uses. In one of the focus groups, it was also pointed out that there is a need to take into account the current uses which, should not be marginalized or phased out due to the development of the practice of bathing and the changes brought about by the public frequenting these places.

•    Trust:

With the co-creation process, the actors of the project indicate that the solutions developed could be suitable for both stakeholders and public, by strategically specifying the content thus, allowing trust in the solutions developed. One participant explained “we believe that if they participated in the development process they would be more willing to use the tool afterwards: they would have trust in it!“ It was important to maintain the “involvement of the participants and make it effective (one must be ready to see the initial plan bifurcate by listening to the actors' concerns and constraints)” and that listening and responding to their concerns was key to maintain the “confidence and involvement in the co-creation process”. Co-creation was necessary because it increases confidence in the solutions. One participant pointed out that “involving the future users in the development of the tool allowed us to create something that perfectly answers their demands, and we were able to build trust in the tool so that they could use it with a lot of confidence”.

•    Governance

Co-creation also raised challenges, first in terms of communication because of the importance of raising awareness among users and populations, and governance. In the Paris case, for example, it allowed for “new questions of governance”, faced political questions to be raised, concerns about swimming in open waters, which technical data to share and to make the decision on the status of a bathing site.

### Format and process

The participants agreed that the co-creation process (format, activities, actors, etc.) implemented was adequate to achieve the objectives.

The **workshop format** was specifically used, a participatory activity where the CoP members were asked for their opinion on potential barriers to the implementation of digital solutions, synergies with their work and their expectations of the DWC project.

In the Berlin case, for example, dedicated workshops with future end-users were held to specify user requirements and test prototypes. This included the ideation of features of the well diary, as well as UX and UI design. This has been essential to support the development of the digital solution and has enabled the DS provider to tailor these products to actual users’ needs. 

In Milan, the third DWC meeting of the CoP had an important focus on co-creation. In this case, a demo version of the serious game on the water, energy, food and climate nexus was presented and discussed with a group of stakeholders. Some educational workshops were also organized. In a similar manner, the DWC Milan leaders have approached several farmers (in collaboration with some of the stakeholders participating in previous CoPs) to directly involve them in the demonstration activities of the matchmaking tool and the related innovations. Again, the reception of this initiative has been very positive and a workshop involving a significant group of farmers and farmers’ representatives was organized.

In the Paris case, INRAE conducted a series of focus groups in order to collect feedback ( Rouillé-Kielo & Bouleau, 2021) from the citizens, organizing a feedback session on all the interviews and focus groups conducted during the project, which were very effective. Several exchange meetings were also organized, with bathing site managers in France sharing their experience in managing a bathing site, a meeting included the exchange of experiences on alternative measurement tools of E.coli that have been used by several institutions.

Some **webinars** were also organized to generate new constructive exchanges. As expressed by one participant: “Great exchange webinar as follow-up action of the CoPs, discussing different techniques and strategies to deal with illicit connections”, for the case of Berlin.

The format offered different options so that everybody had the possibility to meet outside of the main CoPs meeting. In order to understand what kind of format would be the most appropriate, preparations were required. As one participant said, the “key step that enabled the effective calibration of the CoPs was the preliminary individual interviews with the representatives of the invited institutions in order to explain the approach and find out about the institution's position on the subject.

Regarding the format, participants also noted that it was important to “participate without blocking too much of their time so we decided to fix one meeting per month and exchange information via emails”.

**Figure 4.**
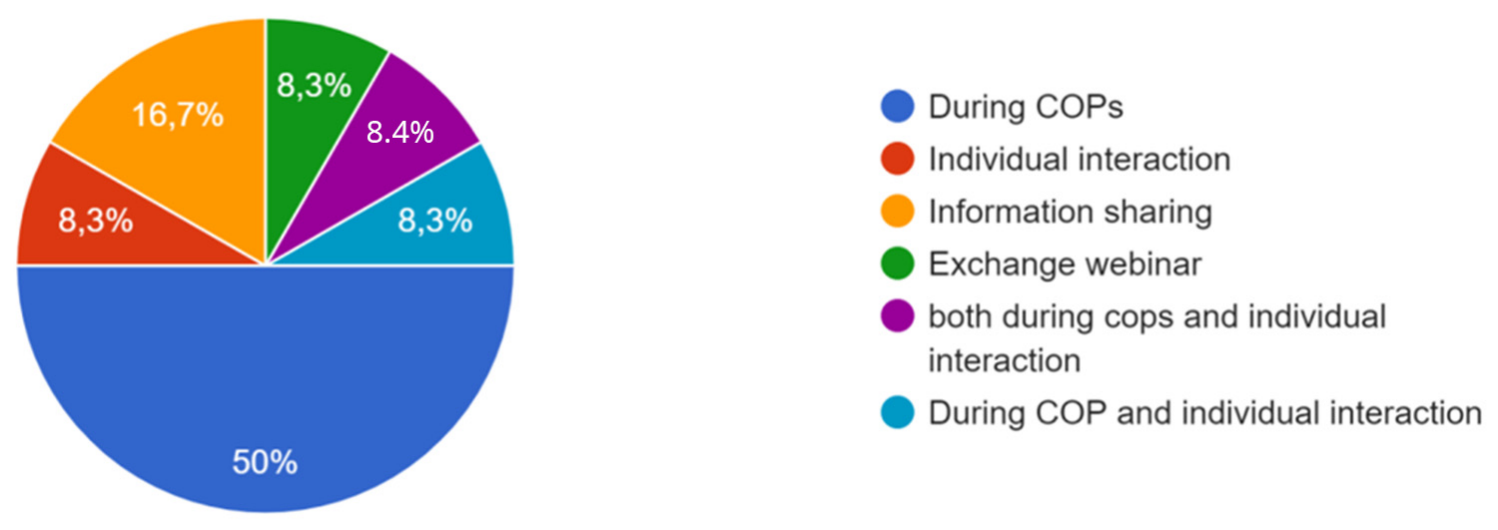
Answers on how the co-creation process occurred.

For the process, co-creation has been carried out, whether at the local level, the city level, the level of project activities, or all three. One participant specified that co-creation was particularly effective at the city level, and that focus groups were added in the methodology. “The intervention of ‘practitioners’ already experienced in CoPs and thus able to share their daily experiences, difficulties and adaptation strategies had a very positive effect on the co-creation process in one of the Local CoPs . In some CoPs, it was the public authorities who were most involved; in other CoPs, it was the innovators, private sector actors and/or researchers. One participant specified that the actors’ involvement also depended on their ‘motivation’ which was influenced by the degree to which the subject affected them. Co-creation processes occurred during the CoPs, as well as through individual interaction and information sharing, as shown the Figure 4.

In the Berlin case, the local CoP raised the question of data exchange between the cities' stakeholders on topics such as groundwater, surface water and rain data. Setting up a common ground in order to work on a secure and centralized data hub for water infrastructure and environmental data will provide a very important foundation for future expansion of the developed digital solutions.

In Copenhagen, the results of the dedicated workshop on the SAMDUS tool have allowed for the integration of key aspects in the solution development roadmap. BIOFOS and DHI have embraced an agile approach for the progress of the SAMDUS web platform. During the workshop, requirements were identified and directly addressed. Beyond this, these requirements were used as criteria for the definition of targets in the development sprints included in the process.

In Milan, a strategy developed during the process was the identification of key "intermediaries" to get in touch with them. Additionally, a collaboration between the Milan municipality and some local high schools was established and a number of educational activities were organized to present the serious game tool and collect some final feedback to be used as direct input for completing the app.

In the Paris case, the fully participatory conception of both applications brought together all the actors working on the bathing situation in the Paris area as well as representatives of all the cities that might open up a bathing site.

### Difficulties and limitations

One of the limitations mentioned was that the process of co-creation “takes a lot of time”. It “took time to set up” and the “co-creation process is quite time intensive”. There was also “time lack for the procedure”. Another difficulty mentioned was to “involve external partners in new processes without funding”. Moreover, some participants indicated that “more results” could be shared in a platform, for example. Another limitation mentioned was that the process could be “closer to the user’s needs”, and that the format could integrate “more physical meetings”, could provide “more structured results from the CoPs”, and integrate other stakeholders, or have “smaller ambitions”.

A difficulty was that “stakeholders in CoP do not have capacity and budget for more involvement in the co-creation”. Some other difficulties were also mentioned, specifically during the online interactive session where some participants debated on modes of communication and political issues and sensibilities, noting that sometimes it was necessary to make a decision and ”not open too much”- also because of budget questions based on specifications.

Another aspect was the importance of “prioritizing”. One participant indicated that to select the features that are relevant to the most end-users, one must be aware of benefits and performance.

**Figure 5.**
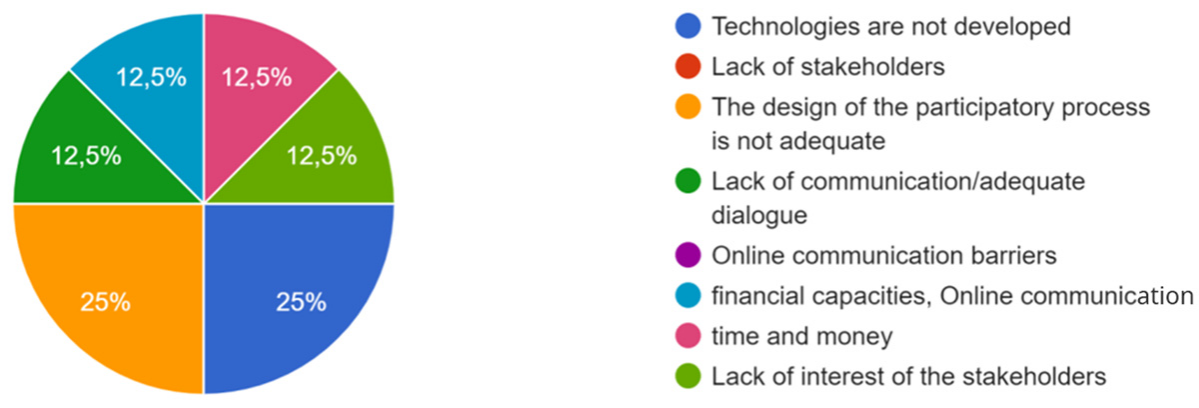
Main causes of barriers when the expected co-creation was not achieved.

When co-creation was not achieved, the main barriers could have different causes, as shown in Figure 5 below.

## Discussion

The methodology proposed by the DWC project for co-creation put in place a process aiming to interact with and facilitate an exchange between different actors, who were encouraged to share their knowledge and resources. Their **regular interactions** made them share “their own resources, integrate resources offered by other actors, and potentially develop new resources through a learning process”, as suggested by the definitions of co-creation in the literature ( Leclercq *et al.*, 2016, p6).

The survey on co-creation within the CoPs and the case studies on the local CoPs showed that one of the objectives, if the context allowed it, to include end-users' needs in the development of the solutions, which is congruent with the literature on co-creation, acknowledging a more constructive role for the customer or end-user ( Galvagno & Dalli, 2014). The necessity of integrating **other stakeholders** into the processes, along with end-users has been underlined as an added value ( Prahalad & Ramaswamy, 2004a). These other stakeholders could include suppliers, public institutions, collaborators. Furthermore, the participative approach of the co-creation process also increases the trust in the solutions developed as the needs and understand and collect expectations.

However, in the Local CoPs, there were some cases where co-creation mostly occurred within companies, because co-creation was not part of the CoP – instead, it occurred internally within these companies as they designed the tools together with users.

The issue of time and effort was not seen the same way by all participants. Some actors of the DWC project highlighted that the process and setting up interactions was taking a lot of time, although other participants expressed that implementing a co-creation process would, from a long term perspective, cost less time and money because the solutions would be better adapted to the needs.

**Regularity** was highlighted as a central element of the process, as well as **adaptability** to the needs of the different actors joining the process.

Regarding the DWC CoPs in general, key issues have been the users’ interest in and relevance of the topics discussed, as well as the subject of the solutions developed. These issues are the foundations of the creation of a community of practice where actors must share a concern or a common subject to constitute the community. Furthermore, as the concept of communities of practice suggested ( Wenger-Trayner, 2015), the “process of collective learning” and sharing mutual knowledge was highlighted by the participants. During the online debate session, it was indicated that one of the central points in constituting the communities was the “ **sense of commitment**”, where several groups of people deliberately choose to engage in regular interactions, common activities and meetings, sharing information and developing skills and solutions together, making them members of a community, as the literature on communities of practice highlighted ( Wenger, 2010).

Finally, another important added value of the co-creation process within the CoPs is that, as explained by some actors, the process would allow them to raise questions in terms of **governance and communication**, and to create new partnerships.

## Conclusions and recommendations

Co-creation was a complex process to set up in order “to establish a culture of co-creation”, adapting the objectives, expectations and format. The role varied depending on the cases and users’ needs. For some Local CoPs, the co-creation was established through a participatory process between private actors, public institutions and researchers. For some other CoPs, the co-creation occurred more directly at a company level, integrating interactions with end-users. End-users’ feedback has been collected, allowing for **greater trust in the solutions** because they are co-created and **adapted to the needs and specificities of the different contexts**.

Co-creation was instrumental in facing some of the sector-specific challenges mentioned before, such as low awareness and engagement of users. As several actors mentioned, the DWC co-creation process and the methodologies of cross-level and cross-sector communities of practice allowed for reflection on policy fit and regulation standards and boundaries.

It could be recommended to add stakeholders and end-users’ feedback once the solutions are fully implemented and operational for some time. It can also be beneficial to include more perspectives and insights during the entire co-creation process, from the very beginning. Including subjective experiences can improve the effectiveness of companies looking to integrate customers (and their employees) into their value processes, as mentioned in the literature ( Galvagno & Dalli, 2014).

## Informed consent statement

This study was carried out during a meeting session with verbal informed consent from all participants. All subjects were the participants and key actors of the DWC project.

## Data Availability

Zenodo: co creation process Answers questionnaire, https://doi.org/10.5281/zenodo.10116376 ( Wehbe, 2022). Data are available under the terms of the Creative Commons Attribution 4.0 International license (CC-BY 4.0).

## Footnotes

^1^This subsection is based on the DWC’s policy brief on the quadruple helix on market opportunities (see Anzaldúa *et al*., 2019), which is accessible at: https://www.digital-water.city/wp-content/uploads/2020/03/DWC_QuadrupleHelixBrief_Final_online.pdf

## References

[ref-1] Andreu L Sánchez I Mele C : Value co-creation among retailers and consumers: new insights into the furniture market. *J Retail Consum Serv.* 2010;17(4):241–250. 10.1016/j.jretconser.2010.02.001

[ref-2] Anzaldúa G Sosa AA Bueb B : Digital Water: outlook and opportunities for academia, business, civil society and public administration.2019. Reference Source

[ref-3] Curley M Salmelin B : Open innovation 2.O: a new paradigm.2013. Reference Source

[ref-4] Carayannis EG Campbell DFJ : ‘Mode 3’ and ‘Quadruple Helix’: toward a 21st century fractal innovation ecosystem’. *Int J Technology Management.* 2009;46(3/4):201–234. 10.1504/IJTM.2009.023374

[ref-5] Chen T Drennan J Andrews L : Experience sharing. *J Mark Manag.* 2012;28(13/14):1535–1552. 10.1080/0267257X.2012.736876

[ref-6] Coviello NE Joseph RM : Creating major innovations with customers: insights from small and young technology firms. *J Mark.* 2012;76(6):87–104. 10.1509/jm.10.0418

[ref-7] Draft policy brief: digitalisation in the water sector. Joint policy recommendations from the DW2020 projects (2022). Author(s): Ulf Stein (DWC), Benedict Bueb (DWC), Andreas Englund (SCOREwater), Richard Eleman (F4W), Natacha Amorsi (F4W), Francesca Lombardo (Aqua3S), Anna Brekine (Naiades), Fernando Lopez Aquillar (F4W), Aitor Corchero (Naiades).

[ref-8] Dige G De Paoli G Agenais AL : Pricing and non-pricing measures for managing water demand in Europe. Technical Report. Service Contract No 3415/B2015/EEA.56130 for the European Environment Agency.2017. Reference Source

[ref-9] European Commission: Water scarcity & drought in the European Union. August 7, 2019.2019. Reference Source

[ref-60] European Environment Agency: Environmental indicator report 2018. EEA Report No 19/2018,2018. Reference Source

[ref-10] European Environment Agency: Water resources across Europe — confronting water stress: an updated assessment. EEA Report No 12/2021.2021. Reference Source

[ref-11] Fuchs C Prandelli E Schreier M : All that is users might not be gold: how labeling products as user designed backfires in the context of luxury fashion brands. *J Mark.* 2013;77(5):75–91. 10.1509/jm.11.0330

[ref-12] Füller J Hutter K Faullant R : Why co-creation experience matters? Creative experience and its impact on the quantity and quality of creative contributions. *R D Manag.* 2011;41(3):259–273. 10.1111/j.1467-9310.2011.00640.x

[ref-13] Füller J Matzler K : Virtual product experience and customer participation - a chance for customercentred, really new products. *Technovation.* 2007;27(6–7):378–387. 10.1016/j.technovation.2006.09.005

[ref-14] Galvagno M Dalli D : Theory of value co-creation a systematic literature review. *Manag Serv Qual.* 2014;24(6):643–683. 10.1108/MSQ-09-2013-0187

[ref-15] Grönroos C : Service logic revisited: who creates value? and who co-creates? *Eur Bus Rev.* 2008;20(4):298–314. 10.1108/09555340810886585

[ref-16] Grönroos C : Conceptualising value co-creation: a journey to the 1970s and back to the future. *J Mark Manag.* 2012;28(13/14):1520–1534. 10.1080/0267257X.2012.737357

[ref-17] Hatch MJ : The pragmatics of branding: an application of Dewey’s theory of aesthetic expression. *Eur J Mark.* 2012;46(7/8):885–899. 10.1108/03090561211230043

[ref-18] Ind N Coates N : The meanings of co-creation. *Eur Bus Rev.* 2013;25(1):86–95. 10.1108/09555341311287754

[ref-19] Lave J Wenger E : Legitimate peripheral participation in communities of practice. Situated learning: legitimate peripheral participation. Cambridge: Cambridge University Press,1991.

[ref-61] Leclercq T Hammedi W Poncin I : Ten years of value cocreation: an integrative review. *Recherche et Applications En Marketing (English Edition).* 2016;31(3):26–60. 10.1177/2051570716650172

[ref-20] Lee L Reinicke B Sarkar R : Learning through interactions: improving project management through communities of practice. *J Proj Manag.* 2015;46(1):40–52. 10.1002/pmj.21473

[ref-21] Li LC Grimshaw JM Nielsen C : Evolution of Wenger's concept of community of practice. *Implement Sci.* 2009;4: 11. 10.1186/1748-5908-4-11 19250556 PMC2654669

[ref-22] López-Gunn E Swinkels J Anzaldúa G : Communities of innovation for climate change adaptation and disaster risk reduction: Niche creation and anticipation. *Sustainability.* 2021;13(9):5180. 10.3390/su13095180

[ref-23] McIntosh A Gebrechorkos SH : Partnering for solutions: ICTs in smart water management. Geneva, Switzerland: ITU,2014. Reference Source

[ref-24] Merz MA Yi H Vargo SL : The evolving brand logic: a service-dominant logic perspective. *J Acad Mark Sci.* 2009;37(3):328–344. 10.1007/s11747-009-0143-3

[ref-25] Nambisan S Baron RA : Virtual customer environments: testing a model of voluntary participation in value Cocreation activities. *J Prod Innov Manage.* 2009;26(4):388–406. 10.1111/j.1540-5885.2009.00667.x

[ref-26] Ostrom AL Bitner MJ Brown SW : Moving forward and making a difference: research priorities for the science of service. *J Serv Res.* 2010;13(1):4–36. 10.1177/1094670509357611

[ref-27] Ostrom AL Parasuraman A Bowen DE : Service research priorities in a rapidly changing context. *J Serv Res.* 2015;18(2):127–159. 10.1177/1094670515576315

[ref-28] Payne AF Storbacka K Frow P : Managing the co-creation of value. *J of the Acad Mark Sci.* 2008;36(1):83–96. 10.1007/s11747-007-0070-0

[ref-29] Plano Clark VL Foote LA Walton JB : Intersecting mixed methods and case study research: design possibilities and challenges. * Int J Mult Res Approaches.* 2018;10(1):14–29.

[ref-30] Prahalad CK Ramaswamy V : Co-creating unique value with customers. *Strategy & Leadership.* 2004a;32(3):4–9. 10.1108/10878570410699249

[ref-31] Riggs W Von Hippel E : Incentives to innovate and the sources of innovation: the case of scientific instruments. *Res Policy.* 1994;23(4):459–469. 10.1016/0048-7333(94)90008-6

[ref-32] Roggeveen AL Tsiros M Grewal D : Understanding the co-creation effect: when does collaborating with customers provide a lift to service recovery? *J Acad Mark Sci.* 2012;40(6):771–790. 10.1007/s11747-011-0274-1

[ref-33] Rouillé-Kielo G Bouleau G : Rendre les cours d’eau urbains baignables, une comparaison Paris-Berlin. PIREN-Seine phase 8 - Rapport 2021. [En ligne],2021. Reference Source

[ref-34] Schau HJ Muñiz AM Jr Arnould EJ : How brand community practices create value. *J Mark.* 2009;73(5):30–51. 10.1509/jmkg.73.5.30

[ref-35] Silva Pinto F Tchadie AM Neto S : Contributing to water security through water tariffs: some guidelines for implementation mechanisms. *J Water Sanit Hyg Dev.* 2018;8(4):730–739. 10.2166/washdev.2018.015

[ref-36] Spohrer J Maglio PP : The emergence of service science: toward systematic service innovations to accelerate co-creation of value. *Prod Oper Manag.* 2008;17(3):238–246. 10.3401/poms.1080.0027

[ref-37] Tajfel H : Differentiation between social groups: studies in the social psychology of intergroup relations.Academic Press,1978. Reference Source

[ref-38] Tajfel H Turner JC : The social identity theory of intergroup behaviour.In: Worchel, S. and Austin, W.G., Eds., *Psychology of Intergroup Relations, 2nd Edition, Nelson Hall, Chicago*,1985;7–24.

[ref-39] Thompson DV Malaviya P : Consumer-generated ads: does awareness of advertising co-creation help or hurt persuasion? *J Mark.* 2013;77(3):33–47. 10.1509/jm.11.0403

[ref-40] Torraco RJ : Writing integrative literature reviews: guidelines and examples. *Hum Resour Dev Rev.* 2005;4(3):356–367. 10.1177/1534484305278283

[ref-41] Tynan C McKechnie S Chhuon C : Co-creating value for luxury brands. *J Bus Res.* 2010;63(11):1156–1163. 10.1016/j.jbusres.2009.10.012

[ref-42] United Nations, Department of Economic and Social Affairs, Population Division: World urbanization prospects: the 2018 revision. Online Edition.2018. Reference Source

[ref-43] Vargo SL Lusch RF : Evolving to a new dominant logic for marketing. *J Mark.* 2004;68(1):1–17. 10.1509/jmkg.68.1.1.24036

[ref-44] Vargo SL : Customer integration and value creation: paradigmatic traps and perspectives. *J Serv Res.* 2008;11(2):211–215. 10.1177/1094670508324260

[ref-62] Vargo S Lusch RF : From products to service: divergences and convergences of logics. *Industrial Marketing Management.* 2008;37:254–259. 10.1016/j.indmarman.2007.07.004

[ref-45] Vargo SL Maglio PP Akaka MA : On value and value co-creation: a service systems and service logic perspective. *Eur Manag J.* 2008;26(3):145–152. 10.1016/j.emj.2008.04.003

[ref-47] Von Hippel E Katz R : Shifting innovation to users via toolkits. *Manag Sci.* 2002;48(7):821–833. 10.1287/mnsc.48.7.821.2817

[ref-48] Water Europe: Water Europe water vision 2030. Brussels: Water Europe,2017.

[ref-63] Wehbe M : co creation process Answers questionnaire. *Zenodo.* [Data set].2022. 10.5281/zenodo.10116376

[ref-49] Wenger-Trayner E : Introduction to communities of practice. A brief overview of the concept and its uses.2015. Reference Source

[ref-50] Wenger E : Communities of practice and social learning systems: the career of a concept. In: Blackmore C, (eds): *Social Learning Systems and Communities of Practice.*Springer, London,2010;179–198. 10.1007/978-1-84996-133-2_11

[ref-51] Zwass V : Co-creation: toward a taxonomy and an integrated research perspective. *Int J Electron Commer.* 2010;15(1):11–48. 10.2753/JEC1086-4415150101

