# Supplementary material for: Insights from the co-creation process in communities of practice for urban water management
Source: Open Res Eur. 2024 Dec 5;4:89. Originally published 2024 Apr 24. [Version 2] doi: 10.12688/openreseurope.16604.2 (PMC11809285; doi:10.12688/openreseurope.16604.2)
Supplement: Peer review history

# Reviewer report: Reviewer response for version 2

**DOI:** 10.21956/openreseurope.20654.r50489

**Published:** 2025-02-24

**Authors:** Love Opeyemi David

**Affiliations:** 1 University of Johannesburg, Johannesburg, South Africa

The study has provided a comprehensive research report on utilizing co-creation and community of practices (CoP) within the EU Digital Water City (DWC) Project. However, there is room for more improvements, given the comments and insights below: 

 The study needs to provide a more detailed statistical account of the participants involved in the five case studies. Specifically, it should include the number of attendees at the workshops, the number of individuals interviewed, and the population and sample size for the quantitative survey, with appropriate scholarly justification. These data should be categorized per case study, following the Bowley proportional allocation system.

 Furthermore, while Tables 1 to 5 highlight various digital innovations across the five case studies, the study should, perhaps in the discussion section, elaborate on how stakeholders navigated the acceptance of these technological innovations for water management in line with the Technology Acceptance Model and the principles of co-creation.  

 Also, following the objectives of Tables 1 to 5, the study should categorically highlight the contribution of co-creation and CoP to the technical performance of the objectives, not just the perception of working together. 

 Moreover, the recommendations section appears shallow, primarily reflecting stakeholder perceptions of co-creation. The recommendations should be presented as a standalone section, drawing directly from the quantitative responses. For instance, the study should offer specific recommendations to address the barriers identified in Figure 5.

If applicable, is the statistical analysis and its interpretation appropriate?

Partly

Is the study design appropriate and is the work technically sound?

Yes

Is the work clearly and accurately presented and does it engage with the current literature?

Yes

Are the conclusions drawn adequately supported by the results?

Partly

Are sufficient details of methods and analysis provided to allow replication by others?

No

Are all the source data and materials underlying the results available?

Partly

Reviewer Expertise:

I am a Research Coordinator at the Center for Cyber-Physical Food, Energy and Water Systems, University of Johannesburg. My research publications and interests center on the Sustainable Security of Water, Energy and Food (WEF) projects, Innovation Management, Management Science and Project Management. Also, I am a management expert on the application of Emerging Technologies such as Artificial Intelligence and Blockchain Technology in WEF Resource management.

I confirm that I have read this submission and believe that I have an appropriate level of expertise to confirm that it is of an acceptable scientific standard, however I have significant reservations, as outlined above.

# Reviewer report: Reviewer response for version 2

**DOI:** 10.21956/openreseurope.20654.r48157

**Published:** 2024-12-27

**Authors:** Teresa Fidélis

**Affiliations:** 1 Department of Environment and Planning, University of Aveiro, Aveiro, Portugal

I believe the improvements introduced in the paper were successful. The paper has clear objectives, methodological steps, and findings. A discussion of the results in the light of the literature could improve further the article. Nevertheless, the overall contents and outcomes are relevant to the scientific community and deserve to be indexed.

If applicable, is the statistical analysis and its interpretation appropriate?

Yes

Is the study design appropriate and is the work technically sound?

Yes

Is the work clearly and accurately presented and does it engage with the current literature?

Yes

Are the conclusions drawn adequately supported by the results?

Yes

Are sufficient details of methods and analysis provided to allow replication by others?

Yes

Are all the source data and materials underlying the results available?

Yes

Reviewer Expertise:

I am a researcher interested in policy and regulatory analysis, and the institutional arrangements for the water circular economy. I recently published an article about the institutional arrangement and water circular economy: https://doi.org/10.2166/wp.2023.155 In addition, I have had an excellent career opportunity as a researcher to work on a European project (Project Ô) focused on integrated water management and the circular economy in four pilots in four countries. Within it, I contributed to problem-solving co-creation processes. A pivotal aspect of the work was problem-solving co-creation process development that employed qualitative methods to address complex challenges (D3.4). Through this process, my colleagues and I facilitated collaborative efforts among diverse stakeholders, fostering innovative solutions that bridged the gap between theory and practice.

I confirm that I have read this submission and believe that I have an appropriate level of expertise to confirm that it is of an acceptable scientific standard.

# Reviewer report: Reviewer response for version 2

**DOI:** 10.21956/openreseurope.20654.r48623

**Published:** 2024-12-27

**Authors:** Alban Kuriqi

**Affiliations:** 1 Universidade de Lisboa, Lisbon, Lisbon, Portugal

Clarity and Focus:

1. Elaborate on the Framework:
  - Expand on what “co-creation” and “Communities of Practice” (CoPs) entail in the context of the DWC project.
  - Define key concepts earlier in the paper to aid readers unfamiliar with these terms.
2. Streamline Objectives:
  - Clarify the specific objectives of the DWC project. The goals of the co-creation process and how they align with the broader project should be explicitly stated.
3. Structure Improvements:
  - Divide the article into clearly defined sections (e.g., Introduction, Theoretical Framework, Methodology, Case Studies, Results, Discussion, Conclusion).
  - Use headings and subheadings to guide readers through the narrative.
4. Details on the Questionnaire:
  - Include more information about the questionnaire used in the study, such as:
    - Design and format.
    - Sample size and demographic details.
    - Key questions or themes addressed.
    - Statistical or qualitative analysis methods applied.

Depth of Analysis:

1. Critical Analysis of Results:
  - Discuss the results in greater depth. For example:
    - What specific needs of end-users were incorporated into digital solutions?
    - Provide examples of how stakeholders collaborated effectively or faced challenges.
2. Address Limitations:
  - Acknowledge limitations of the study (e.g., potential biases, limited generalizability of case studies, or challenges in data collection).
3. Explore Governance Issues:
  - Provide specific examples of the governance questions raised and how they were addressed or remain unresolved.

Contribution and Impact:

1. Highlight Innovations:
  - Emphasize the novel aspects of the co-creation process in DWC compared to other projects or initiatives.
  - Clearly state the added value of the co-creation process for stakeholders, particularly in terms of practical outcomes.
2. Practical Recommendations:
  - Offer actionable recommendations for future co-creation initiatives based on DWC’s successes and challenges.
3. Long-term Impacts:
  - Discuss the potential long-term implications of the partnerships and governance models developed within DWC.

Presentation and Readability:

1. Use Visual Aids:
  - Add diagrams, charts, or tables to summarize the co-creation framework, key findings, or questionnaire data.
2. Improve Language and Flow:
  - Ensure the text flows smoothly by connecting sections logically.
  - Avoid repetition and use active voice where possible to enhance readability.
3. Address Audience Needs:
  - Tailor the content to the intended audience (e.g., academics, policymakers, practitioners) by emphasizing the aspects most relevant to them.

If applicable, is the statistical analysis and its interpretation appropriate?

Partly

Is the study design appropriate and is the work technically sound?

Partly

Is the work clearly and accurately presented and does it engage with the current literature?

Partly

Are the conclusions drawn adequately supported by the results?

Partly

Are sufficient details of methods and analysis provided to allow replication by others?

Partly

Are all the source data and materials underlying the results available?

Partly

Reviewer Expertise:

Floods, Water Resources Managament, Hydraulics

I confirm that I have read this submission and believe that I have an appropriate level of expertise to confirm that it is of an acceptable scientific standard, however I have significant reservations, as outlined above.

# Reviewer report: Reviewer response for version 1

**DOI:** 10.21956/openreseurope.17926.r42025

**Published:** 2024-08-28

**Authors:** Florian Brokhausen

**Affiliations:** 1 Technische Universitat Berlin, Berlin, Berlin, Germany

The paper presents interesting insights from the Digital Water City project. This entails the presentation of five case studies in different European cities, namely Berlin, Paris, Milan, Copenhagen and Sofia. In these cities, so-called Communities of Practice were established to connect a variety of local stakeholders, like end-users, municipalities, tech companies, authorities, etc. The methodology being followed is based on the notion of co-creation, implemented through a variety of different exchange formats. The authors subsequently present the details of how the co-creation process in the Communities of Practice of the five case studies has been established and raise some reflective thoughts as well as achievements and derive recommendations.

 Beside the very relevant and interesting content of the paper, the presentation shows some shortcomings, technical flaws and inconsistencies. The paper needs a structural and linguistic revision. In relation to this, please refer to these general comments:

- Please thoroughly go through the paper again and look for grammatical errors. Especially the omission of articles and prepositions is a prevalent mistake which influences the flow of reading. In addition, there are a lot of convoluted sentences with syntactical errors that should be restructured and can often be simplified. Examples of this are provided below in the minor comments section.
- The structure of the paper makes it hard to follow the train of thought throughout. The Methodology section should be restructured to present specifically, what data is collected, how it is synthesized and how it will serve in answering the three leading questions. With this clear structure established, the outcomes should follow the exact same structure in presenting the data gathered through this process. Subsequently, the discussion reflects upon the contents and findings and answers the leading questions. These three very distinct parts of the paper are currently scattered across these sections and are not well distinguishable.
- The results of the case studies, surveys, interviews and workshops on the different levels of the project are very intertwined, and, therefore, it is often hard to trace in the outcomes where a specific finding originates. As one example, regarding end-user needs in the Paris case study (p.18), the information seems to mainly come from the focus groups that were used, and not from the general meetings on the CoP level as is the case for the other case studies in this section. Therefore, the source of information seems quite different and is not obvious.
- Throughout the outcomes section, there are a lot of very short quotes, presumably from the survey and the interviews. The way these quotes are integrated is rather unconventional, as direct quotes are usually used for important key statements, in contrast to short notions like “took time to set up”, “more results” or “prioritizing”. Please revisit the quotes in this section and evaluate them for necessity.
- The three originally formulated leading questions are never explicitly answered or revisited in the outcomes and discussion. These leading questions could serve as a guiding structure in the composition of the outcomes and discussion which would make it a lot easier to grasp the many findings that are presented.
- The resolution of all figures should be amplified.
- It should be distinguished which (digital) solution was an outcome of the project and the CoPs work itself, and which were pre-existing and have been leveraged or enhanced in this project. As of now, it is not entirely clear, what can be attributed to the DWC project.

Furthermore, here are some detailed, minor comments:

 p.1: “CoPs” is used in the abstract but has not been established as an abbreviation yet. Please spell this out in full.

 p.3: The footnote on the headline of the introduction is bad form. Integrate this information in the text itself.

 p.3: “Concern [in] the water sector is all the more […]” – “in” instead of “around”.

 p.3: “They aim to improve health protection, […]” -> Specify what/who ‘they’ is.

 p.3: The third paragraph presents “Sector-specific challenges”. These could be better presented in a list or enumeration environment to make it more palpable for the reader.

 p.3: “[…] EU Member States, addressing slow innovation rate as well as low awareness […]” -> Either “[the] slow innovation rate” or “slow innovation rate[s]”

 p.3: There are quite some repetitions of the same statements very close to each other, just slightly rephrased, e.g.:

                   "Too little or too much water can have adverse effects on water quality" vs "Both scarcity and abundance of water can have detrimental effects on water quality, [...]"

                   "[...] as well as low awareness and engagement of users, [...]" vs "Finally, users’ awareness and engagement are fundamental points [...]"

 p.3: In the final sentence on the third paragraph, the aforementioned “challenges” are now called “processes”. This is semantically incoherent, please rephrase, e.g. like: “These challenges can be tackled/overcome by […]”

 p.3: In the fourth paragraph the “water value chain” and its different levels are mentioned. Please (briefly) introduce this concept before referring to it and/or provide citations.

 p.4: “Civil society is [a] fundamental actor.”

 p.5: In the caption of Figure 1, the placement of the citation in front of the actual text of the caption is very unintuitive and raises the question how it is related to the subsequent citations within the caption. Please revise.

 p.5: “This can be in regard to a good understanding of the supply and sanitation services and their associated costs, and help highlight their perspectives, needs and point of views.” – What is the ‘their’ related to – the supply and sanitation services, the civil society? Please clarify. Additionally, “perspectives” and “point of views” basically means the same thing.

 p.5: “In the case of DWC, collaboration was facilitated between different actors, such as academics, companies, public administration and end-users, […]” - You can very well explicitly state that these actors were chosen based on the quadruple helix model to represent keys stakeholders; otherwise this statement and choice of actors seems decoupled from the previous explanation of the quadruple helix model.

 p.5: “As mentioned in the co-creation literature, […]” – Please provide the citation needed for this specific mention.

 p.6: “[…] provide relevant solutions towards the digitization of urban water management in European cities.” – This reads more like the appropriate keyword is ‘digitalization’. Please differentiate and use the terms properly.

 p.6: “They are built of public, academic and private stakeholders […]” – What is ‘they’ referring to? Please spell this out.

 p.6: “[…] solutions for water and sanitation infrastructure challenges.” – Please establish a unified wording for this specific application area of interest. Currently, version of the wording are: “water and sanitation infrastructure”, “supply and sanitation services”, “water sector”, “urban water management”, “water treatment and sanitation infrastructure”. A common choice would be to use the term “urban water management” and to establish a proper definition at the start what this term entails.

 p.6: “Local CoPs (level 1), which have been operationalized in four European cities […]” – Why only four, what about Sofia?

 p.6: Figure 2: In the text, the levels are also referred to as level 1, 2, and 3. Please add these indications in the figure as well.

 p.7: The third research question is shorter and differently phrased than in the introduction.

 p.7: “[…] and a workshop mainly involving the pilot and city leaders.” – Please elaborate on the role of the “pilot and city leaders” and what those entail, as they are not mentioned up until this point.

 p.7: “DWC local COPs in action” – So far, the capitalization has always been ‘CoPs’ - please unify the abbreviation.

 p.7: “[…] as shown in […] Table 1, Table 2, Table 3, Table 4 and Table 5.” - Please introduce more of the contents and different sections of the tables, i.e., briefly introduce what information they contain and how this relates to the project. Otherwise, these tables feel very decoupled from the rest of the paper. Also, the subsections in each table often seem to overlap conceptually and content-wise, so an elaboration on the intents of this structure might avoid confusion.

 p.7: “[…] participated in a World Café exercise […]” - For any uninformed reader, please add a citation explaining the format of such a World Café exercise.

 p.8: Table 1: “Berliner Wasserbetriebe” vs. „Berlin Water Works“ – Unify naming.

 p.8: Table 1: „KWB“ - Write this out in full.

 p.8: Table 1: “The CoP worked through meetings and workshops […] and through participatory workshops.” – ‘Workshops’ appears twice in this list.

 p.8: Table 2: “SIAAP, INRAE” - Write these out in full.

 p.8: Table 2: “Expert” and “Public” vs. “expert” and “public” – Unify capitalization.

 p.9: Table 3: “[…] sanitary concerns about water quality.” - As phrased, this is not a goal or an ambition but a concern or challenges. Please rephrase.

 p.9: Table 3: “water reuse-carbon-energy-food-climate nexus” vs. “Water-Energy-Carbon-Climatic nexus” vs. “the link between water, energy, food and climate” – Please refer to the specific nexus of domains consistently.

 p.9: Table 4: “A third solution is a Decision Support System (DSS), […]” - This builds upon the aforementioned solution for flow forecasts I assume? Please mention if so or differentiate clearly if not.

 p.9: Table 4: “The Copenhagen case reunited a group of stakeholders […]” – Reunited - so there was a group/community pre-existing to the DWC project which was reunited for this project? Please elaborate on this.

 p.9: Table 4: “[…] the “Web platform for integrated sewer and wastewater treatment plant control”, which is also directly related to other two DWC solutions […]” – From the phrasing as is, this web platform is presented as something new that was not mentioned in the preceding ‘Digital water innovations supported’ section. Please integrate this properly in the table.

 p.9: Table 4: “HOFOR” - First ever mention of HOFOR in the last section of the table. Please add information on how it relates to the DWC project, the local CoP and add it as a key stakeholder in the table, if appropriate.

 p.10: Table 5: “The objective in Sofia was to create awareness in target audiences about the project objectives and progress.” – This refers to the overall DWC objectives and not the objectives of the local CoP I assume. Please state this clearly.

 p.10: Table 5: “The solution developed was the low-cost temperature sensors for […], which is based on […] low-cost temperature sensors to estimate […]” – This reads as ‘low-cost temperature sensors’ are based on ‘low-cost temperature sensors’ - please rephrase.

 p.10: Table 5: The “key challenges” in the last section of the table, based on content and phrasing, read more like goals and ambitions. Pleas rephrase or restructure.

 p.10: “For some DSs, […]” – This abbreviation has not been used before up until this point, please write out in full.

 p.10: First mention of the abbreviation “ICT”, please write out in full.

 p.11: Figure 3: “Utility” appears twice in the diagram. Additionally, the role of “City Contact” needs to be explained.

 p.18: “More generally on communities of practice, it was said that these mutual learning spaces offered new opportunities for cooperation with other stakeholders.” – This is a repetition of what’s already written in this paragraph.

 p.18: “[…] (for example some aspects were improved, and it is now possible to add new scenarios with few additional developments input).” – This is both too general (“aspects were improved”) and too detailed (“possible to add new scenarios”). Please provide context for the reader.

 p.18: “In terms of expected improvements, the utilities identified several specific attributes, such as scenarios calculation and evaluation, integrated graphs, an overview of different variables, etc.” – Here, some additional details would be interesting, e.g. integrated graphs of what data, what kind of scenarios and which variables.

 p.19: “DS provider” – As above, DS as an abbreviation has not been introduced.

 p.19: “In Milan, the third DWC meeting of the CoP had an important focus on co-creation.” - Co-creation is described to be the focal point of the project and the meetings within the CoPs, so this raises the question, what the focus of the first two meetings was.

 p.19: “For the process, co-creation has been carried out, […]” – What process?

 p.19: “ “The intervention of ‘practitioners’ [..]” – This quote starts but never ends.

 p.20: “[…] the process of co-creation “takes a lot of time”. It “took time to set up” and the “co-creation process is quite time intensive”.” - This is three times the exact same information, no need to insert these quotes.

 p.20: Figure 4: There are again duplicate options. As noted above, “COP” is not consistently capitalized.

 p.21: Figure 5: There are options in the legend that do not appear in the chart. Either somehow signify that they account for 0% or drop them. Further, the capitalization in the legend needs to be unified.

 p.21: “[…] there were some cases where co-creation mostly occurred within companies, because […] – instead, it occurred internally within these companies […]” - Duplicate statement within the same sentence.

 p.21: “It could be recommended to add stakeholders and end-users’ feedback once the solutions are fully implemented and operational for some time.” – I very much disagree with this recommendation and do not think, the results of the project as presented support this conclusion. When a solution is already fully implemented, it is far too late to integrate stakeholders’ and end-users’ feedback.

If applicable, is the statistical analysis and its interpretation appropriate?

Not applicable

Is the study design appropriate and is the work technically sound?

Yes

Is the work clearly and accurately presented and does it engage with the current literature?

Partly

Are the conclusions drawn adequately supported by the results?

Yes

Are sufficient details of methods and analysis provided to allow replication by others?

No

Are all the source data and materials underlying the results available?

Partly

Reviewer Expertise:

I have worked on research regarding various aspects of water and, especially, wastewater infrastructure for several years. In recent years, the focus has shifted to more detailed, technical aspects of wastewater systems and pumping stations. In this line of work, I have been involved in plenty of exchanges with companies in the water and wastewater sector, municipalities, local authorities as well as network and infrastructure operators. Previously, I have been involved in research projects on a European level and have contributed to and authored a variety of publications.

I confirm that I have read this submission and believe that I have an appropriate level of expertise to confirm that it is of an acceptable scientific standard, however I have significant reservations, as outlined above.

# Reviewer report: Reviewer response for version 1

**DOI:** 10.21956/openreseurope.17926.r42024

**Published:** 2024-07-30

**Authors:** Allison Lassiter

**Affiliations:** 1 University of Pennsylvania, Philadelphia, Pennsylvania, USA

It was a pleasure to read about the Digital Water City project and learn about the approach of co-development through communities of practice. To my knowledge, this is a novel method of improving uptake and implementation of digital technologies in water utilities.

 The following revisions will strengthen the paper:

1. There are two core ideas presented within the paper. The first is on digital water -- the development of the COPs and the digital water innovations (e.g., Berliner Wasserbetriebe is interested in AR). The second is the learning during the COP process. The first four paragraphs of the introduction made me think that the intention of the whole paper was to discuss digital water. But, as the paper evolves, it seems digital water is almost ancillary to the questions and instead the paper is really about learning. Please revise the introduction to better frame the main argument.
2. The methods section must be revised, removing all results and enhancing the discussion of the survey:
  - Even if the focus is on learning, there needs to be more background information on the development and engagement of the COPs. Why were these five cities chosen? How were the key stakeholders identified? Exactly how were the COPs established? What is the make-up of the COPs (the number of the people and roles would be helpful)?
  - The numbered lists on pages 7 and 10 should be moved to the Outcomes section.
  - The “Reflecting on the process of co-creation” sub-heading is confusing. This seems like the core method for answering the research questions, but it is buried. Expand on the survey. E.g., how many people was it was distributed to? When? How were the data analyzed?
3. As  a whole, the outcomes section has strong ideas. A challenge, however, is that many of the results are presented by city. Because there is very little discussion of the context of the different cities, it is difficult to understand the value of interpreting the results by city. Please clarify.
4. In the discussion or conclusion, please reflect on the specific value of co-creation and COPs for digital water.

In addition, following are some specific comments:

 p3. The argument presented in the first paragraph could be edited to be shorter and more streamlined. Agriculture is given almost equal weight to cities, which makes it seem like the paper will be about both agriculture and urban areas.

 p3. Eliminating the word “they” will help the text read more smoothly. For example, “They aim to improve health protection…” is more correctly said, “These investments aim to improve heath protection…”. “They” is also used in an uncommon way in the abstract.

 p3. Include a couple sentences explaining why or how digital technologies can help address the issues explained above. The rest of the paragraph addresses shortcomings of digital technologies. Why should digital water technologies be pursued?

 p3. Is there a citation for “Promising solutions often do not reach real applications due to the lack of social and technical preparation, and a mismatch with expectations and needs of end-users”?

 p3. It is good that research questions are clearly stated. There needs to be more background information to understand the questions, however. Define “multi-scale” (what scales?) and “multi-actor” (what actors?). Define what is meant by a “co-creation process”. Is there another way to word question 1?

 p4. The paragraph beginning “The concept has been used…” and the following paragraph seem tangential. Is there a way to relate both more clearly to the research questions? If not, consider considerably shortening the whole section “The concept of value cocreation” and moving this into the introduction before the research questions. Covering this material in the introduction will elevate the concept of co-creation, which is central to the paper’s objectives, and will help orient the reader to what is meant by “co-creation process” before the research questions use this term.

 p6-7. How many people are involved in each COP? Can you give more description of the individuals?

 p7. The research questions do not need to be repeated here. Also, it is unnecessary to broadly justify the utility of a mixed methods approach. Instead, it may be useful to include a brief sentence in the introduction that the study uses a mixed methods approach and implements a survey.

 p7. What is a “World Café exercise”?

 p7. The material under “Based on the discussions, a number of common points were identified…” seems to be a result, rather than a method. It would be clearer to move this from the methods section.

 Table 7. Consider rotating the table to portrait – landscape is a little more difficult to read on a screen and it seems like the table could easily be made portrait.

If applicable, is the statistical analysis and its interpretation appropriate?

Not applicable

Is the study design appropriate and is the work technically sound?

Yes

Is the work clearly and accurately presented and does it engage with the current literature?

Partly

Are the conclusions drawn adequately supported by the results?

Yes

Are sufficient details of methods and analysis provided to allow replication by others?

No

Are all the source data and materials underlying the results available?

Partly

Reviewer Expertise:

Adapting urban water systems to climate change, including evaluating and co-developing digital water solutions.

I confirm that I have read this submission and believe that I have an appropriate level of expertise to confirm that it is of an acceptable scientific standard, however I have significant reservations, as outlined above.

# Reviewer report: Reviewer response for version 1

**DOI:** 10.21956/openreseurope.17926.r40806

**Published:** 2024-06-08

**Authors:** Teresa Fidélis, Fayaz Riazi

**Affiliations:** 1 Department of Environment and Planning, University of Aveiro, Aveiro, Portugal; 2 GOVCOPP and Department of Environment and Planning, University of Aveiro, Aveiro, Aveiro, Portugal

**Your Report**

 Review of the paper

**Insights from the co-creation process in communities of practice for urban water management**

 The paper presents a suitable concept tailored for case studies by adopting the quadruple helix approach for co-creation in the five DWC pilot cities to support the development and potential adoption of innovative digital water solutions. The concepts, including co-creation and communities of practice, are conceptualised and are highly relevant to the co-creation process. The research questions are well-designed and presented in the introduction. Therefore, the paper is well organised and conducted, conceptually guided empirical work. However, some minor revisions are strongly suggested:

- Although the objective is mentioned in the abstract, “ *The article analyzes the ways in which they have been used and applied in DWC as well as the ways in which the project has managed to articulate them at different levels*”, please add it in the introduction before the questions.
- What about Sofia *? Local CoPs (level 1), which have been operationalized in four European cities (i.e. Berlin, Copenhagen, Milan and Paris), mainly focus on the solutions within their local contexts* (page 6). Is Sofia representing another level?
- Please mention how many stakeholders, or respondents are included in the study, in the text.
- Please name table 7 in a complete way, Detailed results of the survey of what and where etc,.
- Please revise the text of Table 7:

- the answer to the first question: The information is confusing. The answers section of question 1, says “ *Local level*”, does it say “Yes, Local level”? If so, please, use a unique way of presenting the answers.
- are the local level and the city level the same? If so, please, use a unique way of presenting the answers.
- *“Yes, in the city and between Paris and Berlin*”, can it be called Project level regarding the definition “ *Project CoP (level 2) provided a useful mechanism to facilitate knowledge exchange between DWC cities (mutual learning) and between DWC cities and technical partners*” (page 6).“ *Not at local level, at project level*”: please clarify this, can be interpreted in many ways, for instance, “Not at the local level, but at the project level”, or “Not at the local and the project level”. It assumed that the answer was “at the project level”. If so, please revise the answers (page 12).

Please remove the reference from the conclusion section *“…into their value processes , as mentioned in the literature (Galvagno & Dalli, 2014)* ”. Considering the authors already supported the including the subjective experience of customers, on page 4.

- Data, I assume that the blank cells in the Excel file mean no answers are available, is that correct?
- Finally, although the study includes five case studies, why the answers received are not case-based? Otherwise, it could have produced better results regarding the various goals and ambitions, innovations supported, and main challenges related to local digital water governance.

If applicable, is the statistical analysis and its interpretation appropriate?

Yes

Is the study design appropriate and is the work technically sound?

Yes

Is the work clearly and accurately presented and does it engage with the current literature?

Yes

Are the conclusions drawn adequately supported by the results?

Yes

Are sufficient details of methods and analysis provided to allow replication by others?

Yes

Are all the source data and materials underlying the results available?

Yes

Reviewer Expertise:

I am a researcher interested in policy and regulatory analysis, and the institutional arrangements for the water circular economy. I recently published an article about the institutional arrangement and water circular economy: https://doi.org/10.2166/wp.2023.155 In addition, I have had an excellent career opportunity as a researcher to work on a European project (Project Ô) focused on integrated water management and the circular economy in four pilots in four countries. Within it, I contributed to problem-solving co-creation processes. A pivotal aspect of the work was problem-solving co-creation process development that employed qualitative methods to address complex challenges (D3.4). Through this process, my colleagues and I facilitated collaborative efforts among diverse stakeholders, fostering innovative solutions that bridged the gap between theory and practice.

We confirm that we have read this submission and believe that we have an appropriate level of expertise to confirm that it is of an acceptable scientific standard, however we have significant reservations, as outlined above.
